# A systematic review of associations between non-communicable diseases and socioeconomic status within low- and lower-middle-income countries

**DOI:** 10.7189/jogh.08.020409

**Published:** 2018-12

**Authors:** Julianne Williams, Luke Allen, Kremlin Wickramasinghe, Bente Mikkelsen, Nia Roberts, Nick Townsend

**Affiliations:** 1 Centre for Population-based Approaches for Non-Communicable Disease Prevention, Nuffield Department of Population Health, University of Oxford, Oxford, UK; 2 Secretariat of the WHO Global Coordination Mechanisms on the Prevention and Control of Non-communicable diseases, World Health Organization, Geneva, Switzerland; 3 Health Care Libraries, Bodleian Libraries, University of Oxford, Oxford, UK

## Abstract

**Background:**

Non-communicable diseases (NCDs) are the leading cause of death globally. Eighty-two percent of premature NCD deaths occur within low- and lower middle-income countries (LLMICs). Research to date, largely drawn from high-income countries, suggests that disadvantaged and marginalized groups have a higher NCD burden, but there has been a dearth of research studying this relationship within LLMICs. The purpose of this systematic review is to map the literature on evidence from LLMICs on the socio-economic status (SES) gradient of four particular NCDs: cardiovascular disease, cancer, diabetes, and chronic respiratory diseases.

**Methods:**

We conducted a comprehensive literature search for primary research published between 1 January 1990 and 27 April 2015 using six bibliographic databases and web resources. We included studies that reported SES and morbidity or mortality from cardiovascular disease, cancer, diabetes and chronic respiratory diseases within LLMICs.

**Results:**

Fifty-seven studies from 17 LLMICs met our inclusion criteria. Fourteen of the 18 papers that reported significant associations between cancer and SES suggested that low SES groups had the highest cancer risk. Eleven of 15 papers reporting significant relationships between CVD and SES suggested that low SES groups have higher risk. In contrast, seven of 12 papers reporting significant findings related to diabetes found that higher SES groups had higher diabetes risk. We identified just three studies on the relationship between chronic respiratory diseases and SES; none of them reported significant findings.

**Conclusions:**

Only 17 of the 84 LLMICs were represented, highlighting the need for more research on NCDs within these countries. The majority of studies were medium to high quality cross-sectional studies. When we restricted our analyses to high quality studies only, for both cancer and cardiovascular disease more than half of studies found a significantly higher risk for those of lower SES. The opposite was true for diabetes, whilst there was a paucity of high quality research on chronic respiratory disease. Development programmes must consider health alongside other aims and NCD prevention interventions must target all members of the population.

**Systematic review registration number:**

Prospero: CRD42015020169.

Non-communicable Diseases (NCDs) disproportionately affect people living within low- and lower middle-income countries (LLMICs) [1-3], with almost three quarters of all NCD deaths and 82% of premature deaths occurring within LLMICs [4]. The relationship between NCDs, poverty and social and economic development has therefore received high-level recognition [5], with NCDs seen to pose a major challenge to development in the 21st century [6,7]. The relationship between social disadvantage and health is complex and shaped by political, social and economic factors [8]. The poor may be more vulnerable to NCDs for many reasons, including material deprivation, psychosocial stress, higher levels of risk behaviour, unhealthy living conditions, limited access to high-quality health care and reduced opportunity to prevent complications [9]. Low socio-economic status (SES) groups are more likely to use tobacco products, consume unhealthy foods, be physically inactive, and overweight or obese [10]. In recent decades, the socio-economic gradient of NCD risk factors has broadened from high-income countries to low- and middle income countries [9,11].

In high-income countries, the evidence suggests that socio-economic factors are positively associated with health [12-16] and that NCDs disproportionately affect lower socio-economic groups [17]. However, there is a lack of research on the relationships between SES and NCDs within developing countries [18-20]. Global cross-country analyses suggest that living in a low- or middle- income country is associated with increased risk of developing cardiovascular diseases (CVD), lung and gastric cancer, type 2 diabetes, and Chronic Respiratory Diseases (CRDs) [20]. These country-level comparisons also indicate that, relative to middle- and high-income countries, low-income countries have some of the highest levels of wealth-related relative inequalities in NCD risk factors [17,21]. However, few studies have considered the relationships between SES and NCDs *within* LLMICs, and it remains unclear whether or not the trends that are observed within higher income countries also hold true within developing countries. The UN’s Sustainable Development Goals for 2030 include a goal “reduce by one third premature mortality from NCDs” (Target 3.4) [22]. In order to work toward this goal, it is important to understand the socio-economic distribution of NCDs within developing countries.

The purpose of this review is to identify, collate, summarize and report the evidence on the intra-*national* distribution of NCDs within LLMICs. Due to the overall lack of primary research from LLMICs and the absence of existing reviews examining the socio-economic distribution of NCDs within developing countries, this review takes a broad approach. It provides a general summary of findings according to region, followed by a detailed discussion of the types of outcomes and types of socio-economic indicators reported.

## METHODS

We included the four outcomes which contribute to the highest NCD burden [23]: cancer, cardiovascular diseases (CVD), chronic respiratory diseases (CRDs) and diabetes. We developed and published a full protocol for this review on the international prospective register of systematic reviews (reference CRD42016039030) [24] and conducted a comprehensive literature search using a combination of free text terms and subject headings to describe socio-economic status and CVD, cancer, diabetes and chronic respiratory disease. We searched the following publication databases: MEDLINE (OvidSP), EMBASE (OvidSP), Global Health (OvidSP), Web of Science Core Collection and Dissertations & Thesis (Proquest). We also included a search of the Global Health Library, restricted to the following regional databases – AIM, LILACS, AMRO, IMSEAR, WPRIM & WHOLIS. Additionally, we hand-searched the reference sections of key articles for additional relevant papers and contacted authors for data that were not reported in published papers. Medline search terms are provided in **Table 1** and full terms and search strategies are provided in Appendix S1 in **Online Supplementary Document(Online Supplementary Document)**.

**Table 1:** Medline search terms

#	Searches
1	cardiovascular diseases/ or heart diseases/ or vascular diseases/ or cerebrovascular diseases/
2	exp Myocardial Ischemia/
3	Heart Failure/
4	exp brain ischemia/ or exp stroke/
5	exp Diabetes Mellitus, Type 2/
6	lung diseases, obstructive/ or exp pulmonary disease, chronic obstructive/
7	exp *Neoplasms/
8	((cardiovascular or cardio-vascular) adj3 disease*).ti,ab.
9	((cardiovascular or cardio-vascular) adj3 (event* or outcome* or risk*)).ti,ab.
10	((coronary or heart or myocard*) adj3 disease*).ti,ab.
11	((coronary or heart or myocard*) adj3 (event* or outcome* or risk*)).ti,ab.
12	((ischaemic or ischemic or ischaemia or ischemia) adj3 disease*).ti,ab.
13	((ischaemic or ischemic or ischaemia or ischemia) adj3 (event* or outcome* or risk*)).ti,ab.
14	myocardial infarct*.ti,ab.
15	((cerebrovascular or vascular) adj3 disease*).ti,ab.
16	((cerebrovascular or vascular) adj3 (event* or outcome* or risk*)).ti,ab.
17	stroke.ti,ab.
18	heart failure.ti,ab.
19	diabet*.ti.
20	((type 2 or type ii or noninsulin dependent or non insulin dependent or adult onset or maturity onset or obes*) adj2 diabet*).ti,ab.
21	(niddm or t2dm or tiidm).ti,ab.
22	(chronic adj2 (lung or pulmonary)).ti,ab.
23	copd.ti,ab.
24	(neoplas* or cancer* or carcinoma* or tumor* or tumour* or malignan* or leukaemia or leukemia or lymphoma?).ti,ab.
25	1 or 2 or 3 or 4 or 5 or 6 or 7 or 8 or 9 or 10 or 11 or 12 or 13 or 14 or 15 or 16 or 17 or 18 or 19 or 20 or 21 or 22 or 23 or 24
26	exp Poverty/
27	Socio-economic Factors/
28	Income/
29	Gross Domestic Product/
30	Economic development/
31	“Salaries and Fringe Benefits”/
32	poverty.ti,ab.
33	((socio-economic or socio-economic or economic) adj2 (factor? or inequalit* or indicator? or status or development)).ti,ab.
34	((household? or house-hold? or family or families) adj3 (income or earning? or wage? or poor or wealth)).ti,ab.
35	(gross domestic product or gross national product or gdp or gnp).ti,ab.
36	(unemploy* or (employment adj2 (status or indicator? or level?))).ti,ab.
37	26 or 27 or 28 or 29 or 30 or 31 or 32 or 33 or 34 or 35 or 36
38	Developing Countries/
39	(Afghanistan or Angola or Armenia or Armenian or Bangladesh or Benin or Bhutan or Bolivia or Burkina Faso or Burkina Fasso or Upper Volta or Burundi or Urundi or Cambodia or Khmer Republic or Kampuchea or Cameroon or Cameroons or Cameron or Camerons or Cape Verde or Central African Republic or Chad or Comoros or Comoro Islands or Comores or Mayotte or Congo or Zaire or Cote d'Ivoire or Ivory Coast or Djibouti or French Somaliland or East Timor or East Timur or Timor Leste or Egypt or United Arab Republic or El Salvador or Eritrea or Ethiopia or Gabon or Gabonese Republic or Gambia or Gaza or Georgia Republic or Georgian Republic or Ghana or Gold Coast or Guatemala or Guinea or Guinea-Bisau or Guam or Guiana or Guyana or Haiti or Honduras or India or Maldives or Indonesia or Kenya or Kiribati or (Democratic People* adj2 Korea) or Kosovo or Kyrgyzstan or Kirghizia or Kyrgyz Republic or Kirghiz or Kirgizstan or Lao PDR or Laos or Lesotho or Basutoland or Liberia or Madagascar or Malawi or Nyasaland or Mali or Mauritania or Micronesia or Moldova or Moldovia or Moldovian or Mongolia or Morocco or Ifni or Mozambique or Myanmar or Myanma or Burma or Nepal or Netherlands Antilles or Nicaragua or Niger or Nigeria or Pakistan or Palestine or Papua New Guinea or Paraguay or Philippines or Philipines or Phillipines or Phillippines or Rwanda or Ruanda or Samoa or Samoan Islands or Navigator Island or Navigator Islands or Sao Tome or Senegal or Sierra Leone or Sri Lanka or Ceylon or Solomon Islands or Somalia or Sudan or Swaziland or Syria or Principe or South Sudan or Tajikistan or Tadzhikistan or Tadjikistan or Tadzhik or Tanzania or Timor-Leste or Togo or Togolese Republic or Uganda or Ukraine or Uzbekistan or Uzbek or Vanuatu or New Hebrides or Vietnam or Viet Nam or West Bank or Yemen or Zambia or Zimbabwe or Rhodesia).hw,kf,ti,ab,cp.
40	((developing or less* developed or under developed or underdeveloped or low* middle income or low* income or underserved or under served or deprived or poor*) adj (countr* or nation? or state? or population? or world)).ti,ab.
41	((developing or less* developed or under developed or underdeveloped or low* middle income or low* income) adj (economy or economies)).ti,ab.
42	(low* adj (gdp or gnp or gross domestic or gross national)).ti,ab.
43	(lmic or lami).ti,ab.
44	transitional countr*.ti,ab.
45	38 or 39 or 40 or 41 or 42 or 43 or 44
46	exp Mortality/
47	Morbidity/
48	Survival Rate/
49	(mortality or survival or death?).ti,ab.
50	morbidit*.ti,ab.
51	46 or 47 or 48 or 49 or 50
52	25 and 37 and 45 and 51
53	cardiovascular diseases/mo or heart diseases/mo or vascular diseases/mo or cerebrovascular diseases/mo or exp Myocardial Ischemia/mo or Heart Failure/mo or exp brain ischemia/mo or exp stroke/mo or exp Diabetes Mellitus, Type 2/mo or lung diseases, obstructive/mo or exp pulmonary disease, chronic obstructive/mo or exp *Neoplasms/mo
54	53 and 37 and 45
55	52 or 54

We included papers published between January 1, 1990 and 27 April 2015. Papers were required to provide outcome data on cardiovascular diseases, cancer, diabetes, or chronic respiratory diseases (CRDs) occurring within LLMICs, which were defined according to the World Bank’s 2013 definition (per capita income of less than or equal to US$ 1045 for LICs and between US$ 1046-US$ 4125 for LMICs) [25].

To compare outcomes between different socio-economic groups, we required papers to report NCDs stratified by an indicator of SES (see **Table 2**) and to include an internal comparison between groups. To include research from as many LLMICs as possible, we did not restrict papers based on study design, type of SES indicator or by the summary analysis of the outcome measure.

**Table 2:** Population, exposure, comparator and outcome for the systematic review

Population	Exposure	Comparator	Outcome
General populations (different income levels) from low- and lower middle-income countries [25]	Socio-economic status, measured according to income, access to basic needs (eg, housing), human capabilities (eg, literacy), household possessions/wealth or other measures.	We compared outcomes among populations from one socio-economic level to those in another	Morbidity or mortality (including premature mortality) from NCDs from:
	In the initial screening, we did not restrict studies based on the type of SES or poverty indicator that the paper used.	Where possible, we focused on outcomes among the lowest SES groups and by sex	1) Cardiovascular diseases (coronary heart diseases and strokes)
			2) Cancer
			3) Diabetes (type II)
			4) Chronic respiratory diseases

### Identifying papers for inclusion

JW examined the titles, abstracts and full-text articles, bringing uncertainties to LA, KW, and NT. A second researcher (LA) checked a 10% sample of titles and full text articles. The percentage agreement was calculated and deemed acceptable using Cohen's Kappa statistics [26]. Disagreements were resolved via group consensus. Heterogeneity in exposure and outcome measures precluded meta-analysis, so we adopted a narrative approach that summarised studies by disease outcome and WHO region.

### Quality assessment

Study quality relates to the appropriateness of its design, implementation, analysis and presentation for a given research question [27]. There were no existing quality assessment tools that were appropriate for all of the papers in this review, so we adopted a descriptive approach for quality assessment using a modified version of the Newcastle-Ottawa scale [28,29] and methods presented by Herzog et al [30]. The tool implements a points-based system to judge the quality of studies based on the selection of the study groups, the comparability of the groups and the ascertainment of the measures. We used these scores to rank study quality as “high”, “medium” or “low” quality and stratified them according to their study design.

## RESULTS

**Figure 1:**
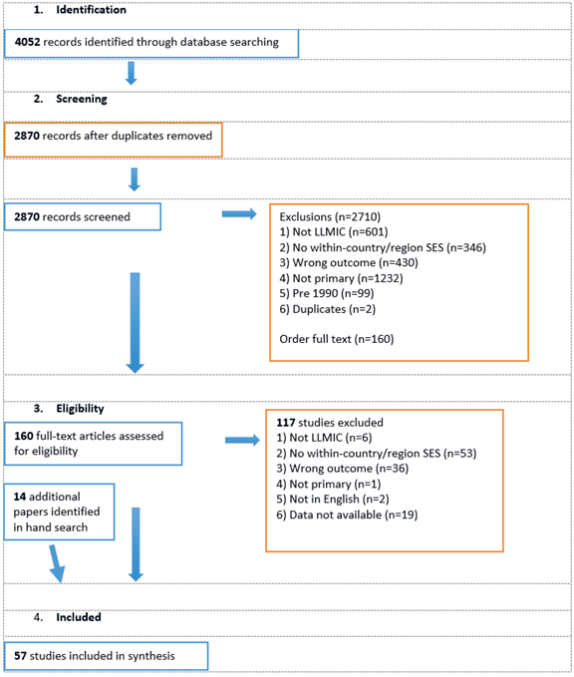
Summary of search information.

**Figure 2:**
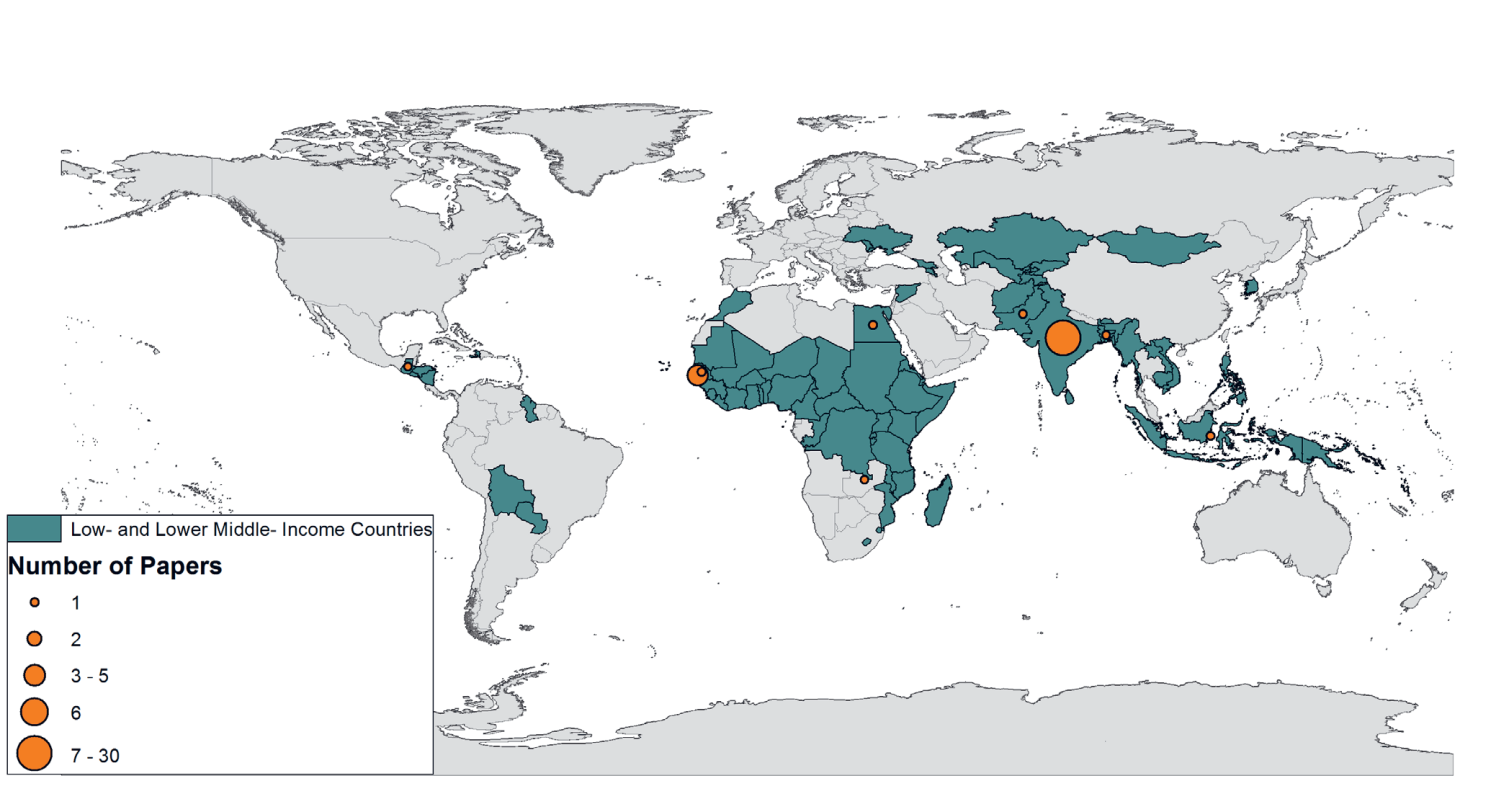
Map of low- and lower middle-income countries represented by research papers in this review.

**Figure 3:**
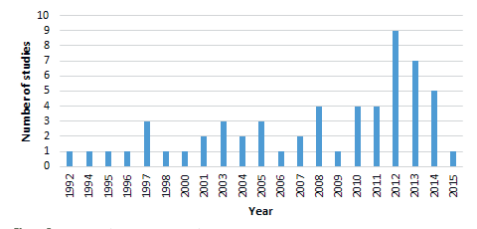
Number of studies by year of publication.

The search information is summarised in **Figure 1** following PRISMA guidelines [31]. More than 4000 articles were retrieved by the initial search and an additional 14 were found via hand searching. A total of 57 studies from 17 countries and presenting outcomes from 494 904 people were included in the final review (**Figure 2**). Around half (n = 27) of the studies included in their abstract an explicit aim to investigate associations between SES and NCDs, while the remaining studies aimed to investigate a wide range of factors, including SES. Around half (n = 30) published within the last six years (**Figure 3**). Thirty of the studies were cross-sectional, 18 were cohort studies, and nine were case-control studies. Around half of the studies in this review used population-based surveillance systems or household sampling methods to identify and recruit study participants from the community while the remaining half drew study participants from patient populations in hospital-based studies. We ranked 26 of our studies as “high” quality, 28 as “medium” quality and 3 as “low-quality”. The quality of papers was limited by weaknesses related to selection of controls, ascertainment of exposures/outcomes, and high non-response rates. For a detailed description of the quality assessment scores, please see Appendix S2 in **Online Supplementary Document(Online Supplementary Document)**. We included eight papers from Africa, seven from the Eastern Mediterranean, six from the Western Pacific, three from the Americas and one from the European Region. The most well represented region was Southeast Asia, which contributed 32 papers, of which 27 came from India.

### Types of outcomes

Summaries of included studies, grouped according to outcome and region, are provided in **Tables 3.1-3.5** and full study results are provided in Appendix S4 in **Online Supplementary Document**. Of the four conditions that we considered, the most frequently reported outcome was cancer (n = 21) (most commonly of the breast (n = 8) or cervix (n = 4)). The second most common outcome was cardiovascular disease (n = 20), which included AMI (n = 4), stroke (n = 4), ischemic heart disease (n = 1), angina (n = 1), coronary heart disease (n = 1), peripartum cardiomyopathy (n = 2), and CVD as one aggregated group (n = 3). Twelve papers included diabetes as an outcome. Two studies specified insulin-dependent diabetes and the remaining nine did not define type. Among papers that reported the method of diabetes assessment, two papers used self-report to determine diabetes status, while five specified the use of biochemical parameters. Three papers reported outcomes related to CRDs. One study looked exclusively at chronic obstructive pulmonary disease, while the remaining two studies considered respiratory diseases (type not specified) as one of several outcomes reported alongside other conditions. Four papers included in this review reported an aggregated NCD measure that included a wide range of conditions.

### Measures of socio-economic status

Thirty-five studies used education-based measures of SES (eg, literacy or years of schooling), 22 used an aggregate SES score (based on a combination of education, income, housing, social castes or standardized scales), and 11 used an income-based measure. Appendix S3 in **Online Supplementary Document(Online Supplementary Document)** provides a detailed summary of reported measures of socio-economic status.

### Associations between non-communicable diseases and socio-economic status

**Figure 4:**
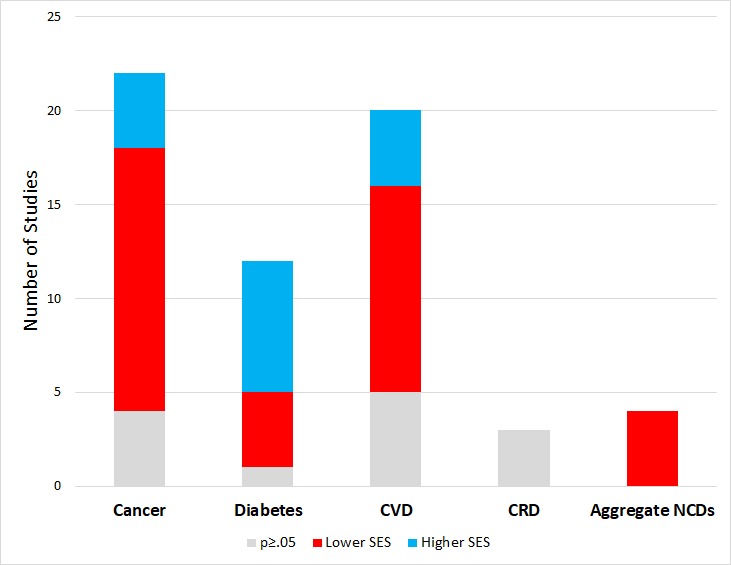
Summary of reported associations between socio-economic status (SES) and non-communicable diseases (NCD) outcomes.

Thirty-three of the 48 significant associations that were reported among papers in this review placed lower SES groups in the highest risk category for NCDs, but the pattern was not uniform across disease categories (**Figure 4**).

### Cancer

This review included 22 papers on cancer. Eighteen papers reported significant associations, with fourteen of these suggesting that lower SES groups have higher cancer risk, the remaining four suggested that higher income groups have a significantly higher risk of cancer (**Table 3**).

**Table 3:** Included studies with cancer outcomes, by region

Study	Country	Design	n	Quality	Sample	Exposure	Outcome	Highest Risk Group
**Africa**								
Jordan et al (2013) [32]	Tanzania	Case-control	345	Medium	115 cases and 230 age- and district-matched controls	Property Index	Breast cancer	Higher property level* (A)
Misganaw et al (2013) [33]	Ethiopia	Cross-sectional	3,709	Medium	Malignancy mortality data for 3709 adults (15 and older) who died in Addis Ababa between 2006 and 2009 (identified via burial surveillance) and whose families could be interviewed to determine cause of death	Education	Malignant neoplasm mortality	Higher education† (A)
Parkin et al (2000) [34]	Uganda	Case-control	194	High	Cases included 50 adults and 132 children with histologically diagnosed lymphoma. Controls were adults with cancers unrelated to HIV and children with non-infectious diseases	Education	non-Hodgkin lymphoma	Higher education
Aziz et al (2008) [35]	Pakistan	Cohort	525	Medium	525 patients with stage I-III breast cancer presenting to the Department of Oncology from January 1997 to December 2005	SES	Event free survival in stage I-III breast cancer	Lower SES‡ (A)
							Overall survival in stage I-III breast cancer (n = 525)	Lower SES† (A)
Aziz et al (2010) [36]	Pakistan	Cohort	237	High	237 women undergoing multimodality treatment for locally advanced breast cancer (LABC) treated between Jan 200 and December 2005 at Jinnah Hospital Pakistan)	SES	Event-free survival in locally advanced breast cancer	Lower SES* (A)
							Overall survival in locally advanced breast cancer	Lower SES
Aziz et al. (2004) [37]	Pakistan	Cohort	286	High	286 patients with breast cancer recruited between 1996 and 1998	SES	Breast cancer	
							Size of tumour (71)	Low SES†
							Stage at diagnosis (I)	Low SES†
							Mean number of lymph nodes positive	Low SES
							Mean time elapsed before diagnosis (months)	Low SES†
							Mean age at diagnosis (years)	Low SES†
							Five year disease free survival	Low SES‡
							Five year overall survival	Low SES†
Chaouki et al (1998) [38]	Morocco	Case-control	460	High	245 cases and 215 controls from Rabat Morocco. Cases of invasive cervical cancer identified among first attendants at hospital. Cases were verified histologically. Controls identified at same hospital and in a nearby general hospital	Education	Cervical cancer	Lower education‡
						Wealth (facilities at home)		Fewer facilities at home‡
						Income		Lower family income‡
Khan et al (2015) [39]	Pakistan	Cross-sectional	315	Low	315 female patients from Oncology Institute in Islamabad	Education	Breast cancer presentation delay	Lower education *
						SES		Lower SES*
Bonilla et al (2010) [40]	El Salvador	Cohort	886	High	433 de novo acute lymphoblastic leukaemia patients 0-16 y of age, diagnosed between 2000 and 2007	Education (parent)	Event free survival (standard risk patients)	Lower education (parent)† (A)
						Income	Event free survival (High risk patients)	Lower monthly income* (A)
						Education (parent)	Event free survival (standard risk patients)	Lower education (parent) (A)
						Income	Event free survival (High risk patients)	Lower monthly income (A)
Gavidia et al (2012) [41]	El Salvador	Cohort	251	High	251 children aged 0-16 y with newly diagnosed acute leukaemia treated at Benjamin Bloom hospital, San Salvador	Mother's education	Leukaemia patient outcome: Sepsis	Mother illiterate* (A)
						Father's education		Father literate (A)
						Income		Annual Household Income<US$ 2000 (A)
						Household Characteristics		Clean water at home (A)
						Household Characteristics		No toilet at home (A)
								
						Mother's education	Leukaemia patient outcome: Infectious death	Mother illiterate (A)
						Father's education		Father illiterate (A)
						Income		Annual Household Income<$2000* (A)
						Household Characteristics		No clean water at home (A)
						Household Characteristics		No toilet at home (A)
								
Gupta et al (2012) [42]	El Salvador, Honduras, Guatemala	Cohort	279	High	279 patients younger than 21 y diagnosed with AML from 2000 to 2008 in El Salvador, Honduras or Guatemala	Monthly purchasing power units	Leukaemia: treatment-related mortality	Higher Purchasing Power (A)
						Education (parent)		Higher education (A)
Ali et al (2008) [43]	India	Cross-sectional	522	Medium	Breast cancer patients from Kerala or Tamil Nadu visiting a regional cancer centre	SES	Late stage diagnosis of breast cancer (stages III and IV)	Lower SES (A)
						Education (parent)		Illiterate* (A)
Burkart et al (2011) [44]	Bangladesh	Cross-sectional	21,551	Medium	Mortality data (n = 21 551) from the Bangladesh Bureau of Statistics for period from 2002 to 2007, using a sample comprised of 1000 primary sample units in rural and urban areas. Deaths recorded by officials quarterly and then verified through field visits	SES (of region)	Deaths attributable to cancer	High SES region
Chankapa et al (2011) [45]	India	Cross-sectional	968	Medium	968 adult women aged 15-60 y	Education	Cervical cancer: Low-grade squamous intraepithelial lesion	Illiterate
						Monthly family income	Cervical cancer: High-grade squamous intraepithelial lesion	Lower income
Dutta et al (2005) [2]	India	Cohort	121	Medium	Between 1994 and 2001, 121 patients diagnosed with gallbladder cancer were evaluated prospectively in the gastroenterology services of two hospitals	SES	Age of diagnosis for gallbladder cancer	Low SES* (A)
Gajalakshmi et al (1997) [3]	India	Cohort	1747	High	1747 patients registered at population based cancer registry (part of national network) (inclusion criteria: follow up data available)	Education	Five year breast cancer survival (% by education level)	Illiterate* (A)
Mostert et al (2012) [46]	Indonesia	Cohort	143	High	145 patients diagnosed with a malignancy at academic hospital (Inclusion criteria required age to be between 0-16 y with newly diagnosed malignancy	Hospital class, insurance status	Childhood cancer (Abandonment of treatment)	Poor†
							Childhood cancer (Event-free survival)	Poor†
Patil et al (2014) [47]	India	Case-control	380	High	141 cases of patients with hepatocellular carcinoma and 240 controls with chronic liver disease (age 18-70), seeking treatment at two centres in Mumbai	Income	Hepatocellular carcinoma	Higher income*
Sankaranarayanan et al (1995) [48]	India	Cohort	452	High	Cervical cancer cases (age 35-65) registered from 1 January to 31 December	SES	5 y survival from cervical cancer	Low SES* (A)
Ngaon et al (2001) [49]	Vietnam	Cross-sectional	5034	Medium	5034 patients with cervical cancer treated in the Central Oncology of Ho Chi Minh City from Nov. 1989-Nov 1994	Education	Cervical cancer	Illiterate*
Lan et al (2013) [50]	Vietnam	Cohort	948	High	5034 patients with cervical cancer treated in the Central Oncology of Ho Chi Minh City from Nov. 1989-Nov 1994	Education	Survival probability following diagnosis of breast cancer	Lower education* (A)
							Survival probability following diagnosis of breast cancer (Extended Cox model assessing effect)	Lower education* (A)
Xin et al (2014) [51]	Mongolia	Cross-sectional	643	Medium	643 deaths	Education	Years of life lost (YLL) due to colorectal cancer and average years of life lost (AYLL) due to colorectal cancer in each region	Higher education†

A – adjusted, y – years, SES – socio-economic status

*<0.05.

†<0.01.

‡<0.001.

Thirteen papers examined associations between cancer and education. Ten papers found significant relationships, eight of which reported a higher risk among the least educated. These included studies from India [3,43], El Salvador [40,41], Morocco [38], Pakistan [39], and Vietnam [49,50]. Two medium quality cross-sectional studies reported significant positive associations between education and cancer, with the more highly educated showing a greater number of years of life lost due to colorectal cancer in Mongolia [51] and higher mortality from malignant neoplasms in Ethiopia [33]. Three studies did not find significant associations between education and cancer, representing research from El Salvador, Honduras and Guatemala [42], Uganda [34] and India [45].

Among the five studies that examined associations with cancer and income [38,40,41,45,47], three reported that low-income groups had significantly higher risk [38,40,41]. Having a low income was associated with lower rates of event free leukaemia survival in El Salvador [40], increased odds of cervical cancer in Morocco [38], and prolonged waiting times for assessment and treatment, sepsis and infectious death among leukaemia patients with paediatric fever in El Salvador [41]. A fourth study reported that low-income patients had a higher risk of cervical cancer in India, but this was non-significant [45]. Contrary to the other studies, a fifth paper reported that higher-income patients had a significantly higher risk of hepatocellular carcinoma in India [47].

Among 13 studies that examined associations between cancer and SES as an aggregate measure or some other measure of wealth, eleven reported higher risk among those falling into lower SES. The association was significant for eight [2,32,36-39,46,48] and non-significant for two [41,43].The remaining study reported a non-significant association in the opposite direction, with a higher number of deaths attributable to cancer among those living in a high SES region compared to a low SES region [44].

Restricting findings to high quality papers, five of seven papers reporting on education and cancer found that the lower educated were more likely to get cancer or have poor cancer outcomes [3,38,40,50,52]. Three [38,40,41] of four high quality studies reporting on income and cancer found that those with lower incomes were more susceptible to cancer (and this was significant for two) [40,41] but not for the third [38]. There were two high quality studies reporting on cancer and wealth and both found that those with lower wealth had worse outcomes [38,41]and this was significant for one [41].

### Cardiovascular disease

We included 19 studies that reported CVD outcomes. Among the nine studies that examined CVD and education, six found significant relationships and for five of these studies, the highest risk was observed among those with lower education [52-56]. The remaining two found that compared to those with lower levels or no education CVD risk was greatest among those with at least some education[57] or those with higher levels of education [58] (**Table 4**).

**Table 4:** Included studies with cardiovascular disease outcomes, by region

Study	Country	Design	n	Quality	Sample	Exposure	Outcome	Highest risk group
**Africa**								
Miszkurka et al (2012) [59]	Burkina Faso	Cross-sectional	,822	Medium	4822 adults over the age of 18 (47% male) sampled through a multi-stage stratified random cluster sampling strategy by gender, age, rural/urban setting)	Education	Angina (prevalence)	Some education (A)
						Income		Higher income* (A)
**Eastern Mediterranean**									
Engels et al (2014) [60]	Morocco	Cross-sectional	44 742	Medium	Data from 13 279 households (60 031 individuals) collected via door-to-door survey. Sampled population demographically and socio-economically representative of Morocco	Wealth	Stroke prevalence (overall)	Lower wealth (A)
						Wealth	Stroke prevalence (rural)	Medium-levels of wealth (A)
						Wealth	Stroke prevalence (urban)	Lower wealth* (A)
**South East Asia**									
Burkart et al (2011) [44]	Bangladesh	Cross-sectional	21 551	Medium	Mortality data from the Bangladesh Bureau of Statistics for period from 2002 to 2007 in rural and urban areas. Deaths recorded by officials quarterly and then verified through field visits	SES (of region)	Deaths attributable to CVD	High SES region†
Chandrashekhar et al (2014) [57]	India	Cross-sectional	370	Low	370 persons age 60 and older in urban and rural field practice area of Department of Community medicine in Gulbarga	Education	CVD	Some education†
						Class	CVD	Middle class
Das et al (2007) [61]	India	Cohort	51 533	High	52377 adults living in Kolkata, selected via stratified random sampling	Slum vs non-slum	Stroke prevalence	Residents of slum areas
							Stroke incidence	Residents of slum areas
							Stroke (case fatality rate)	Residents of non-slum areas
Dogra et al (2012) [62]	India	Case-control	442	Medium	442 participants (184 cases (≤40 y) with definite AMI (as per WHO criteria) and 350 (≤40 y) controls)	SES	AMI	Low SES† (A)
Kisjanto et al (2005) [58]	Indonesia	Case-control	917	Low	235 cases and 682 age-matched controls of women (aged 20-44 y) from 14 hospitals in Jakarta, recruited between 1989 and 1993	Social class	Stroke	Higher class (A)
						Education	Stroke	Higher education* (A)
Singh et al (1997) [63]	India	Cross-sectional	1764	High	Adults living in randomly selected villages who had lived in the area since birth	SES	CAD (males) (Crude)	High SES†
						SES	CAD (females) (Crude)	High SES*
						SES	CAD (males)	High SES (A)
						SES	CAD (females)	High SES (A)
Xavier et al (2008) [64]	India	Cohort	18 862	High	12 405 patients given a definitive diagnosis of electrocardiograph changes or suspected MI who were readmitted 30 d later	Class	Odds of mortality after 30 d following acute coronary symptoms (CRUDE)	Poor* (A)
						Class	Odds of mortality after 30 d following acute coronary symptoms (Adjusted)	Poor (A)
Yadav Ret al (2013) [65]	India	Cohort	599	High	599 stroke registry patients from urban and rural population admitted to the neurology department at a tertiary care centre in North India	Job status	Stroke morbidity	Lower occupation category (A)
Gupta et al (1994) [52]	India	Cross-sectional	3148	High	3148 residents aged over 20 (1982 men, 1166 women) divided into various groups according to years of formal schooling. Randomly selected from a cluster of three villages in rural India	Education	CHD (men)	Lower education (men)
						Education	CHD (women)	Lower education (women)†
Pednekar et al (2011) [66]	India	Cohort	148 173	High	148 173 individuals aged ≥35 y were recruited in Mumbai during 1991-1997 and followed to ascertain vital status during 1997-2003	Education	CVD mortality (men)	Less education (A)
						Education	CVD mortality (women)	Some education (A)
Rao et al (2011) [56]	India	Cross-sectional	2129	Medium	Sample of adults with CVD (n = 2129) and diabetes (439) drawn from 47 302 rural and 26 566 urban households	Education	CVD (self-reported, including hypertension)	Lower education*
Rastogi et al (2011) [55]	India	Case-control	1050	High	350 cases and 700 controls, recruited equally from New Delhi and Bangalore. The subjects’ mean (SD) age was 52 (11) years, and 12% of the subjects were women	Education	AMI (relative risk)	Lower education* (A)
						Income	AMI (relative risk)	Lower income* (A)
Pais et al (1996) [67]	India	Case-control	400	High	200 Indian patients with a first acute myocardial infarction (AMI) and 200 age and sex matched controls aged 30-60years	Income	AMI (Odds if low income)	Low income* (A)
Singh et al (2005) [68]	India	Cross-sectional	2222	Medium	2842 randomly selected adults (25-64) who died between July 1999 to July 2001 in Moradabad, India	Social Class 1 (More Affluent)	Circulatory diseases (mortality)(males)	Higher social class*
Sharieff et al (2003) [69]	Pakistan	Cohort	35	Medium	35 patients with heart failure in the last month of pregnancy or 5 mo postpartum	SES	Peripartum cardiomyopathy: Recovery or deterioration after 6 mo	Lower SES*
**Western Pacific**									
Hoang et al (2006) [54]	Vietnam	Cohort	1067	High	1067 adults (≥20 y) living in a predominantly rural area died of all causes from 1999-2003	Education	CVD mortality	Less education* (A)
						SES	CVD mortality	Non-poor (A)
Minh et al (2003) [70]	Vietnam	Cohort	64	High	249 deaths (64 related to CVD) registered in Bavi District, a population of approximately 50000 in a rural area 60 km to the west of Hanoi	Education	CVD mortality	Inconclusive (A)
						Economic condition	CVD mortality	
Enkh-Oyun et al (2013) [53]	Mongolia	Cross-sectional	2280	Medium	2280 people aged over 40 y from eight rural slums (counties villages) using WHO STEPs surveillance manual	Education	Ischemic heart disease (IHD) prevalence (overall)	Lower education (overall) (A)
						Education	IHD prevalence (men)	Some education (men) (A)
						Education	IHD prevalence (women)	Lower education (women)† (A)

SES – socio-economic status, IHD – ischemic heart disease, CVD – cardiovascular disease, AMI – acute myocardial infarction, CAD – coronary artery disease, A – Adjusted, y – years, mo – months, d – days

*<0.05.

†<0.01.

‡<0.001.

Three papers investigated associations between income and CVD [55,59,67] and in two, lower income groups had significantly higher risk of AMI [55,67]. In the third study, higher income groups had a significantly higher prevalence of angina [59].

Eleven studies measured associations between SES and CVD and five reported significant findings after adjusting for confounders [44,60,62,63,68,69]. Among three, CVD risk was highest among low SES groups. Lower SES was significantly associated with stroke prevalence among urban dwellers [60], AMI and risk of poor outcomes from peripartum cardiomyopathy [69].

Overall, 11 papers reported significant findings suggesting that lower SES groups had higher CVD risk, including seven studies from India, [52,55-57,62,64,67] two from the Eastern Mediterranean countries of Morocco [60], and Pakistan [69], and two from the Western Pacific countries of Mongolia [53] and Vietnam [54]. By contrast, four papers reported significant findings suggesting that higher SES groups had worse CVD outcomes, representing findings from three Southeast Asian countries (Bangladesh [44], Indonesia [58], India [68], and Burkina Faso [59]). We did not observe clear patterns according to disease subtype or region, although India was the one country from which studies reported significant findings in both directions, with six studies indicating that lower SES groups had significantly higher CVD risk [52,55,56,62,64,67] and one indicating that higher SES groups had significantly greater CVD risk [68]. That said, these studies were conducted in a range of regions throughout India using many different outcomes, exposures, study designs and populations. For example, Singh (2005) examined social class and mortality from circulatory diseases in Moradedabab in 2005 using a cross-sectional study design, while Dogra examined associations between AMI and SES in Chandigarh using a case-control study in 2012. Xavier examined mortality rates following coronary symptoms and class using a prospective cohort in 10 different regions and Pais examined AMI and SES using a hospital-based case-control study in Bangalore in 1996.

Four high quality papers reported on education and CVD and in three, those with lower levels of education had worse outcomes [52,54,55]. One high quality paper reported on associations between wealth and CVD and was not conclusive. Five high quality studies reported on SES and CVD and in two, those with lower SES had worse outcomes [63,70].

### Diabetes

We included twelve studies that reported diabetes outcomes. Overall, four papers reported significant findings which placed lower SES groups in the higher diabetes risk category, while seven reported significant findings placing higher SES groups in the higher risk category (**Table 5**).

**Table 5:** Included studies with diabetes outcomes, by region

Study	Country	Design	n	Quality	Sample	Exposure	Outcome	Highest risk group
**Africa**								
Bella et al (1992) [71]	Nigeria	Case-control	57	Medium	Insulin-dependent ketosis prone diabetics seen over a period of six years at University College hospital	Social class	Diabetes	Lower social class†
						Education		Lower education†
						Employment status		Non-skilled†
Fekadu et al (2010) [72]	Ethiopia	Case-control	217	Medium	107 cases and 110 controls from two regions recruited from diabetic clinics. Age and sex controls were recruited from general medical clinics for conditions other than diabetes in both communities	Occupation	Diabetes	Unskilled worker† (A)
						Education		Lower education† (A)
						Presence of animals		Animals sleeping in same room (A)
						Access to toilet		No access to toilet† (A)
						Access to water		No access to piped/clean water† (A)
						Hatched (vs corrugated) roof		Hatched Roof (A)
						No. of people/house		More people in the house (A)
						No. of people/room		More people sleeping in one room* (A)
						Distance from clinic, km		Greater Distance From Clinic† (A)
						Possessions index		More Possessions† (A)
Ploubidis et al (2013) [73]	Kenya	Cross-sectional	4314	High	4314 participants ≥50 y of age	Wealth	Diabetes (type not specified)	Wealthier* (A) (Rural)
						Wealth		Wealthier* (A) (Urban)
**South East Asia**								
Safraj et al (2012) [74]	India	Cross-sectional	78 173	Medium	78 173 rural adults 35 and above	SES	Diabetes (type not specified), SR	Higher SES* (higher prevalence among SES Group 4 in all groups) (A)
Reddy et al (2007) [75]	India	Cross-sectional	31 866	Medium	19 973 individuals 20-69 y of age	Education	Diabetes (type not specified)	Some education (A) (Men)
								Lower Education† (A) (Women)
Corsi et al (2008) [76]	India	Cross-sectional	2439	Medium	168 135 survey respondents aged 18–49 y (women) and 18–54 y (men)	Caste	Diabetes (self-report)	Scheduled caste (A)
						Wealth		Richest* (A)
						Education		Some education
Sayeed et al (1997) [77]	Bangladesh	Cross-sectional	2362	High	1052 subjects from urban and 1319 from rural communities (age ≥20 y) of different socio-economic classes were investigated	Wealth	Non-insulin dependent diabetes mellitus	Richest* (A)
Gupta et al (2003) [42]	India	Cross-sectional	1123	High	1123 adults (>20 y living in urban India)	Education	Diabetes (self-report)	Some education (men)
								Some education (women)
Ajay et al (2008) [78]	India	Cross-sectional	10 930	High	10 930 urban adults (age 20-69)	Education	Diabetes (diagnosed according to fasting glucose)	Lower education‡ (A)
Kinra et al (2010) [79]	India	Cross-sectional	1983	Medium	1983 adults (aged 20-69 y) living in rural areas from 18 states in India	SES	Diabetes (based on self-report and blood glucose)	High SES† (A) (Men)
								Middle and high SES (A) (women)
Samuel et al (2012) [80]	India	Cross-sectional	228	High	2218 adults (age 26-32) from urban and rural setting involved in a cohort study (birth years: 1969-73)	SES	Diabetes (blood glucose diagnosis)	Highest SES† ((A)) (male, urban)
								Middle SES ((a)) (female, urban)
								Highest SES‡ (a) (male, rural)
								Highest SES‡ (a) (female, rural)
						Education		Highest education (a) (male, urban)
								Highest education (a) (female, urban)
								Highest education (a) (male, rural)
								Highest education (a) (female, rural)
Zaman et al (2012) [81]	India	Cross-sectional	4535	High	4535 adults (aged 30+) recruited from rural Andhra Pradesh (mean age 49.4, SD 13.6)	Education	Diabetes (type not specified)	Educated† (A) (Male)
								Educated (A) (Female)
						Occupation		Skilled‡ (A) (Male)
								Skilled (A) (Female)
						Income		High (A) (Male)
								High† (A) (Female)
Rao et al (2011) [56]	India	Cross-sectional	2129	Medium	Sample of adults with CVD (n = 2129) and diabetes (439) drawn from 47 302 rural and 26 566 urban households	Education	Diabetes (self-reported)	Lower education*

SD – standard deviation, CVD – cardiovascular disease, SES – socio-economic status, A – adjusted

*<0.05.

†<0.01.

‡<0.001.

Eight studies considered associations between education and diabetes and five reported significant results. In four of these, a lower education was significantly associated with diabetes in the overall study populations in Nigeria and Ethiopia [71,72] and in two among women only in India [56,75]. In contrast, one Indian study reported a significant association between a higher level of education and diabetes risk among women, but not men [81].

Ten studies considered associations between SES as an aggregate measure, class, occupation level or wealth and nine of these presented significant results. In seven of these, the higher SES groups were at significantly greater risk of diabetes, including studies from India [74,79-81], Bangladesh [77] and Kenya [73]. In contrast, two studies found that diabetes risk was greater among lower class or non-skilled workers in Nigeria [71] and lower skilled workers or people lacking access to water and toilet facilities in Ethiopia [72].

Restricting to high quality studies only, the three high quality studies reporting on associations between diabetes and education found that those with high levels of education had worse outcomes [73,80,81]. The paper by Zaman et al was the only high quality study reporting on associations between income and diabetes and found that high-income women had the worst outcomes. Two high quality studies reported on wealth and diabetes and in both, the wealthier and highest income had the worst outcomes [73,80]. Similarly, the four high quality papers on SES and diabetes also found that those with the highest SES had the worst outcomes [73,77,80,81].

### Chronic respiratory diseases (CRD)

Three cross-sectional studies from Southeast Asia [44,57,82] examined associations between SES and CRDs. A study from Bangladesh analysed all-cause and cause-specific mortality by age, gender and socio-economic condition in urban and rural areas. While the study reported significant differences in risk of dying from CVD in high SES regions compared to low SES regions, there were no significant differences in the risk of dying from respiratory diseases. A second study from India examined morbidity patterns among urban and rural geriatric populations and associations with education and SES and found that there were no significant differences between education groups or socio-economic classes in respiratory disease[57]. Finally, a cross-sectional study of ambient air pollution and chronic respiratory morbidity was conducted in Delhi and found no significant differences in the prevalence of chronic bronchitis or COPD by SES [82] (**Table 6**).

**Table 6:** Included studies with chronic respiratory disease outcomes, by region

Study	Country	Design	n	Quality	Sample	Exposure	Outcome	Highest risk group
**SE Asia**								
Burkart et al (2011) [44]	Bangladesh	Cross-sectional	21 551	Medium	Mortality data (n = 21 551) from the Bangladesh Bureau of Statistics for period from 2002 to 2007, using a sample comprised of 1000 PUSs in rural and urban areas. Deaths recorded by officials quarterly and then verified through field visits.	SES (of region)	Deaths attributable to Respiratory disease (%)	High SES
Chhabra et al (2001) [82]	India	Cross-sectional	4171	Medium	Permanent residents of Delhi who were 18+ years of age and living near one of nine permanent air quality-monitoring stations.	SES	COPD/chronic bronchitis	Lower SES (in both higher and lower pollution zones)
Chandrashekhar et al (2014) [57]	India	Cross-sectional	370	Low	370 persons age 60 and older in urban and rural field practice area of Department of Community medicine in Gulbarga.	Education	Respiratory disease	Some education
						SES		Middle/lower class

COPD – chronic obstructive pulmonary disease, SES – socio-economic status

### Multiple NCDs

Four cross-sectional studies [12,83-85] reported associations between SES and NCDs as an aggregate measure rather than by specific diseases and three of them found significant relationships that identified lower SES individuals at greater risk of NCDs. In a study from Kosovo, those who perceived themselves as poor were more likely to report having chronic conditions and multi-morbidity [12]. Similarly, Vietnamese women with a lower education and lower SES were more likely to self-report chronic conditions [84]. Finally, in a cross-sectional study from Burkina Faso, adults with lower education and lower standards of living had significantly greater risk of mortality from NCDs [85]. A cross-sectional study from India reported a non-significant association between lower income status and self-reported NCDs [83] (**Table 7**).

**Table 7:** Included studies with multiple non-communicable disease outcomes, by region

Study	Country	Design	n	Quality	Sample	Exposure	Outcome	Highest risk group
**Africa Region**								
Rossier et al (2014) [85]	Burkina Faso	Cross-sectional	409	Medium	Adults (≥35 y) living in formal and informal neighbourhoods of Burkina Faso who died between 2009 and 2011. There were 409 deaths among 20 836 study participants	Education	Mortality From NCDs (CVD, Neoplasms, Other NCDs, Asthma, Diabetes, Liver Disease)	Lower education* (A) (in both formal and informal neighbourhood’s)
						Standard of living		Lower standard of living* (A) (in both formal and informal neighbourhoods)
**European Region**								
Jerliu et al (2013) [12]	Kosovo	Cross-sectional	1890	Medium	A representative sample of 1890 individuals aged ≥65 y (949 men, mean age 73 ± six years; 941 women, mean age 74 ± 7 y	Education	Presence of self-reported chronic diseases (CVD, diabetes, stomach and liver, lung, neurologic disorders, cancer and other conditions)	No education (A)
						Self-perceived poverty		Poor† (A)
						Education	Multi-morbidity (2 or more chronic conditions)	No education (A)
						Self-perceived poverty		Poor* (A)
**South East Asia**								
Binnendijk et al (2012) [83]	India	Cross-sectional	38 205	Medium	Rural populations from two states in India	Income	Self-reported NCDs (The three most prevalent were musculoskeletal, digestive and cardiovascular problems)	Lower income* (Region of Odisha)
								Lower income (Region of Bihar)
**Western Pacific**								
Van Minh et al (2008) [84]	Vietnam	Cross-sectional	2484	Medium	2484 adults aged 25-74 selected using stratified random sampling for personal household interview	Education (Males)	Self-reported chronic diseases (including chronic joint problems, heart and circulatory conditions, cancer, diabetes, chronic pulmonary diseases and psychological illness)	Lower education
						SES (Males)		Middle SES
						Education (Females)		Lower education*
						SES (Females)		Lower SES*

NCD – noncommunicable disease, CVD – cardiovascular disease, SES – socio-economic status, A – adjusted

*<0.05.

†<0.01.

### Description of findings by region

#### Africa

Three studies on cancer came from Africa and two reported significant results: Having a higher level of property was associated with breast cancer in Tanzania [32] and higher education was associated with malignant neoplasm mortality in Ethiopia [33]. Two of the three African diabetes studies from Nigeria and Ethiopia found that low SES was associated with diabetes [71,72], while one Kenyan study found that the wealthier participants had higher risk [73]. One cross-sectional study in Burkina Faso found higher angina prevalence among those with a higher income [59].

#### Eastern Mediterranean Region

Lower SES groups had worse cancer outcomes according to four studies from Pakistan [35-37,39]and one from Morocco [38]. Another study from Morocco reported associations between lower wealth and stroke [60].

#### Region of the Americas

Two studies from El Salvador found that lower SES groups had worse outcomes [40,41], but a third study found that higher SES groups had higher treatment related mortality [42].

#### Southeast Asia

There were varying results in Southeast Asia: higher SES was associated with coronary artery disease [63] and higher class was associated with circulatory diseases [68]. Higher SES was associated with CVD deaths in Bangladesh [44]. However, in India, lower SES was associated with acute myocardial infarction (AMI) [62], mortality following acute coronary symptoms [64] and AMI [67]. Lower education was associated with coronary heart disease among women [52] and AMI [55]. In Pakistan, low SES was associated with peripartum cardiomyopathy [69]. High SES was associated with diabetes in India [74,79,80] and Bangladesh [77], but also with lower education levels[75,78].

#### Western Pacific

Higher education was associated with years of lost life due to colorectal cancer in Mongolia [51], but lower education was associated with IHD among women [53]. In Vietnam, lower education was associated with CVD mortality [54] and lower SES was associated with multiple chronic conditions [84].

#### Europe

In Kosovo, one paper reported an association between poverty and the presence of self-reported chronic conditions [12].

## DISCUSSION

This review systematically mapped the literature on associations between SES and NCDs from 57 papers within 17 LLMICs. In summary, 14 of the 18 papers with significant associations between cancer and SES suggested that lower SES groups have higher cancer risk. Eleven of the 15 papers reporting significant relationships between CVD and SES suggested that lower SES groups have higher risk. In contrast, seven of 11 papers with significant findings related to diabetes and SES found that higher SES groups had higher risk. Our findings are consistent with a review by Hosseinpoor, which also found that unlike other NCDs, higher diabetes prevalence occurred among the wealthier and more educated, especially in low-income countries [17]. Sommer et al conducted an overview of systematic reviews on socio-economic inequalities and NCDs. Unlike our study, this study compared outcomes between countries (rather than individuals within countries). However, similar to our findings related to cancer and cardiovascular disease, Sommer et al found that having low SES or living in an LMIC increased the risk of developing CVD, lung and gastric cancer and increased the risk of mortality from lung cancer and breast cancer. Unlike our study, it found that having low SES also increased the risk of developing type 2 diabetes or COPD and increased the risk of mortality from COPD [20]. Allen et al conducted a review on the association between SES and NCD risk factors (harmful use of alcohol, tobacco use, unhealthy diets, and physical inactivity within LLMICs). Just as we found that low SES groups had worse outcomes related to cancer and CVD, Allen et al found that low SES groups had a significantly higher prevalence of tobacco and alcohol use, and consumed less vegetables, fish and fibre [21]. However, the results related to SES and NCD risk factors from Allen et al were inconsistent; the review also found that high SES groups were less physically active and consumed more fats, salt, and processed foods compared to low SES groups.

There are multiple reasons why more advantaged groups in LLMICs may be at a higher risk of some NCDs. For example, according to the nutrition transition theory, increasing wealth is often associated with shifts in dietary and physical activity patterns, which may lead to the predominance of nutrition-related NCDs [86]. Reported socio-economic inequalities in NCDs may be biased by differential access to health care services between different SES groups [87]. It is possible that NCD cases among low SES groups were more likely to be under-diagnosed, leading to an underestimation of the true prevalence rates. These variations highlight a need to identify and implement effective development strategies that reduce the overall burden of disease without increasing health inequalities in LLMICs. These findings also demonstrate the importance of setting up national-level surveillance systems to examine the relationship between SES and NCDs using a nationally representative sample.

We observed wide between-study variation in terms of study populations, NCD outcomes, SES measures and study designs. We chose broad inclusion criteria to ensure that we could identify relevant research from as many LLMICs as possible. Given the lack of previous reviews on this issue and, relative to research from high-income countries, the scarcity of primary research articles reporting on NCDs and SES within these countries, we chose to err on the side of inclusion, despite the heterogeneity that such an approach might cause. That said, applying a more inclusive approach towards selection of studies in terms of exposure also poses a limitation on this study. Different measures of SES capture different underlying dimensions of a person’s position in society [88]. For example, current income allows researchers to measure access to material goods and services that may influence health, but this measure is age dependent and more unstable than education or occupation. Wealth tends to be more strongly linked to social class than income, and having assets often corresponds with an ability to meet emergencies or to absorb economic shock. Education is a fairly stable measure beyond early adulthood and is likely to capture aspects of lifestyle and behaviour, but it has different social meanings and consequences in different contexts, economic returns may differ significantly across groups, and SES does not rise consistently with increases in education years. Given these differences, choosing the best variables for measuring SES should depend on consideration of the likely causal pathways and relevance of the indicator for the populations and outcome under study [88]. The causal pathway between SES and NCDs is complex, so it is challenging to identify ‘the best’ indicator to use when examining associations between SES and NCD outcomes. This highlights a need for more research, not only on how various SES indicators are associated with health outcomes, and how the relationship changes depending on the indicator, but also more research on the theoretical aspects of NCDs and the hypothesized causal pathways between SES and disease.

Previous studies on NCDs and LLMICs have largely focused on global cross-country comparisons. To the best of our knowledge, this is the first review to focus exclusively on the distribution of NCDs *within* LLMICS. Additional strengths include our comprehensive search strategy and adherence to PRISMA guidance.

Our search strategy was restricted to English search terms, which may have limited representation of research from LLMICs. We assessed study quality using the Newcastle Ottawa scale because it allowed us to assess the quality of nonrandomised studies, but previous studies have reported low inter-reliability using this method[89] [90]. These scores should not be taken to assess the quality of the work outside of the context of this review, given that many studies had aims that extended beyond the investigation of associations between SES and outcomes.

Our search strategy was also restricted to research from 1990-2015. We limited our study to cover research from 1990-2015 due to the high volume of search results associated with having multiple outcomes and multiple exposure measurements, coupled with resource restraints. Rather than starting at a later time, we chose to start in 1990 because this was the year that the Millennium Development Goals were signed, and when targets and indicators to monitor progress (including many socio-economic indicators) started to become more widely integrated into research studies [91]. Future research may benefit from considering associations between NCDs and SES over a longer time period.

Our search strategy (**Table 1**) included a range of exposure terms related to poverty, socio-economic factors, income, gross domestic product, household wealth, wages, poverty, wealth and employment. However, it did not include the term ‘social class.’ The results from the search included many papers that used the terms we’d identified alongside the term ‘social class’ to describe the exposure variable. We decided to include these papers in our review, despite the fact that ‘social class’ was excluded from the initial search method, because this term that was often used interchangeably with the other SES terms. However, this decision may have biased the results by excluding other papers on social class which were overlooked by our search strategy.

This review relied on evidence from resource-scarce research environments that lack the infrastructure required to collect reliable morbidity and mortality statistics on a routine basis. Around half of our studies collected data from hospitals, research environments that are associated with well-documented limitations [48]. In addition, the data we present from surveillance systems may also have limitations. According to Das et al, in 1994 only 13.5% of all deaths in India were medically certified [61], and there are likely to be associations between a person’s SES status and medical certification at death. Lozano et al also highlights regional heterogeneity and the need for sound epidemiological assessments of causes of death [92]. Many of the included studies had small sample sizes that were not nationally representative. We included these studies in order to highlight research from as many LLMICs as possible, but this decision also severely limits the generalisability of our findings. For this reason, we included three ‘low quality’ studies in our final analysis. Two were related to cardiovascular disease and one was related to cancer. Excluding these studies from our final analysis did not change the overall trends in our results. Nine of 13 papers reporting significant relationships between SES and CVD suggested that low SES groups have higher risk. Thirteen of the 17 papers that reported significant associations between cancer and SES suggested that low SES groups had the highest cancer risk. The relationship related to diabetes risk remained unchanged, with seven of the 11 papers reporting significant findings related to diabetes finding that higher SES groups have higher diabetes risk.

About half of the studies included in this review were conducted in hospital settings and there may be a bias related to the high socio-economic profile of hospital patients, which may not represent the population characteristics of a country. Therefore, we conducted additional analyses to explore whether or not associations between SES and NCDs differed depending on the setting. The results are as follows. For cancer, 15 of the 18 studies reporting significant associations were hospital-based studies. Of these 15 hospital-based studies, 13 reported that low SES individuals had higher cancer risk, while two reported that high SES individuals had higher risk. Of the three community-based studies, two reported that higher SES individuals had higher risk and one reported that lower SES individuals had higher risk. For cardiovascular disease, six of the 15 papers reporting significant results were hospital-based studies. Among these six hospital-based studies, five reported that low SES individuals had higher risk of cardiovascular disease. Of the nine community-based studies, four reported that low SES individuals had higher cardiovascular disease and five reported that high SES individuals had higher risk. Of the 12 papers reporting significant associations between diabetes and SES, three were hospital-based and nine were community-based. All three of the hospital-based studies found that low SES groups had higher diabetes risk. Seven of the nine community-based studies found that high SES groups had higher risk and two found that low SES groups had higher risk.

We observed that the magnitude and direction of associations between NCDs and SES may vary according to the type of NCD, but our findings also suggest that the direction of the association may also depend on the type of SES indicator, which corroborates earlier findings [93]. There are advantages and disadvantages to the indicators used by studies in this review, discussed elsewhere in the literature [94,95].

Access to health care and knowledge about disease conditions is associated with wealth and education [13,87,96,97], which may lead to biased results when examining associations between SES and health. Vellakkal et al determined whether socio-economic inequalities in the prevalence of NCDs differed if estimated by using symptom-based measures compared with self-reported physician diagnoses [87]. They found that SES gradients in NCD prevalence tended to be positive for self-reported physician diagnoses but were attenuated or became negative when using symptom- or criterion-based measures, particularly in low-income countries.

Low-SES NCD patients are particularly vulnerable; People in many LLMICs must finance health care through out-of-pocket payments, which places a burden on NCD patients and families [56,98,99]. The recurring nature of these costs [83], coupled with the reduced productivity that accompanies poor health [100] causes additional problems.

This review included only three articles with CRD outcomes, and none of them reported significant findings. Two of these articles did not describe the diagnostic modality. One limitation to this study is that in an attempt to include research from as many LMICs as possible, we did not limit inclusion based on diagnostic modality. Chest x-rays, spirometry, CT scans and arterial blood gas analysis are all used for diagnosis, but these require access to health care facilities, which may be limited among low SES groups. It is likely that there is a high prevalence of undiagnosed COPD. Gribsby et al examined the association between SES and COPD prevalence using data collected in Argentina, Bangladesh, Childe, Peru and Uruguay and found that adjusted odds ratio of having COPD was lower for people who completed secondary school and lower among those with higher monthly household incomes [101]. This finding is compatible with other research which has found that poor populations tend to have a higher risk of developing COPD and its complications than their wealthier counterparts [102-104]. A recent Global Burden of Disease study examined how sociodemographic development has a different effect on the burden of COPD and asthma and show that mortality but not prevalence of asthma is strongly related to sociodemographic development [105].

In conclusion, this review highlights the need for more research on the associations between NCDs and SES within a wider range of LLMICS; there were 67 LLMICs from which we did not identify any publications eligible for inclusion. We encourage more research on NCD morbidity and mortality in LLMICs [106], along with the collection and presentation of SES indicators alongside these NCD measures. Lack of NCD surveillance data presents a challenge to the prevention and control of NCDs in low- and middle-income countries. Many countries lack the resources to develop and maintain an information system to collect, analyse and disseminate data and information on trends in NCDs and socio-economic status [107]. The WHO STEPwise approach to risk factor surveillance to assess NCDs and their risk factors provides one relatively low-cost option for monitoring within-country trends, but also for making comparisons across countries [108]. The STEPwise approach to risk factor surveillance survey includes several questions on socio-economic status. While it may not be possible to find one single measure of SES that is universally relevant across all study contexts, having more studies integrate some of these WHO measures into their reporting would strengthen the evidence base about relationships between SES and NCDs.
