# Supplementary material for: A systematic review of associations between non-communicable diseases and socioeconomic status within low- and lower-middle-income countries
Source: J Glob Health. 2018 Jul 25;8(2):020409. doi: 10.7189/jogh.08.020409 (PMC6076564; doi:10.7189/jogh.08.020409)
Supplement: Online Supplementary Document [file jogh-08-020409-s001.zip › Appendix_3_SES Measures.docx]

**Appendix 3.** Types of exposure measures from included studies

| **Study** | **Indicator of socio-economic status** |
| --- | --- |
| Ali et al. (2008) | Household Income (low, med, high) "assessed by social workers based on a variety of factors" based on occupation, partner, ownership /rental of land. |
| Aziz et al. (2004) | SES (high, middle, low) < $500 USD = low, $500-1000 = middle, $>1000=high) |
| Aziz et al. (2008) | SES (poor, middle, high). SES was evaluated according to family income (low SES <$200/month, middle: $200-$500/month, high: >$500 month). |
| Aziz et al. (2010) | SES (low, middle) was assessed using median family income (low: up to $11/day, middle: between $11 and $25/day), high: greater than $25 per day). |
| Bella et al. (1992) | Education (completed primary, completed secondary, attending polytechnic/university, graduated from polytechnic/university), Employment status (nonskilled, skilled, unemployed) |
| Binnendijk et al., (2012) | Income-proxy (monthly per capita consumer expenditure excluding healthcare costs). Amounts were reported in Indian rupee and then converted into international dollars for purchasing power parity (PPE). |
| Bonilla et al., (2010) | Parental education (Illiterate, Primary, Secondary, Advanced) |
| Burkart et al., (2011) | Socio-economic status (high or low) at the level of the administration (subunit of 6 divisions within Bangladesh. SES was based on child mortality rate, child/woman ratio, literacy rate, fertility rate, source of drinking water, infant mortality, insolvency rate and use of solid fuels) |
| Chandrashekhar et al., (2014) | Education status (Illiterate, Primary, Secondary, College, Graduation, Post Graduate, Total), Socioeconomic class (I, II, III, IV and V) |
| Chankapa et al., (2011) | Educational status (Illiterate, Class≤ V, ≤ VIIII, ≤ X, ≤ XII), Per capita monthly family income in Rupees (≤500, 501-1000, 1001-1500, 1501-2000, 2001-2500, Total %) |
| Chaouki et al., (1998) | Education (none, primary, secondary, higher), Number of facilities at home (running water, toilet, TV, refrigerator, telephone, video, stereo set, motorbike, car); Family income (high, middle, low) |
| Chhabra et al., (2001) | SES based on monthly income (in U.S. dollars): (1) lower SES-income below $1 00.00; (2) middle SES-income between $1 00.00 and $350.00; and (3) higher SES-income above $350.00. |
| Corsi et al., (2008) | SES defined as social caste, education, household wealth |
| Das et al., (2007) | Slum areas versus non-slum areas (X2 comparisons); Education (Primary- standard I to X; Higher- standard XI and higher); Income (per family per month: Rs 2500 (US$ 56), Rs 2500–5000 (US$ 56–111) Rs 5000 (US$ 111)) (No significance testing for differences between SES groups. |
| Dogra et al., (2012) | SES (high/middle/low) |
| Dutta et al., (2005) | SES defined according to the modified Kuppuswamy scale, which has been standardized for Indian patients |
| Engels et al., (2014) | Wealth index: Multiple Correspondence Analysis (MCA) was used to construct wealth index based on 15 variables: type of housing, number of rooms, type of lighting, source of water supply, presence of housing amenities (kitchen, bathroom and toilet), and the household’s possession of durable assets (television, satellite dish, phone and cell phone, stove, fridge, washing machine, and car). |
| Enkh-Oyun et al., (2013) | Education (categorized per the Mongolian system into 5 categories: elementary (7 years), incomplete secondary (7–9 years), complete secondary (10–12 years), vocational (13, 14), and university graduates (> 14 years). Monthly income (lower, middle, and upper) levels according to the average salaries per month (Mongolian National Statistic Office 2009); Socioeconomic position was measured and defined as social classes based on the longest held occupation, using a Registrar General’s Classification (Class I corresponded to the upper social class. Participants were placed into 6 groups of social class I (professional, e.g., physicians, engineers), social class II (managerial e.g. Teachers, sales managers, social class III (semi-skilled non-manual, e.g., bricklayer), social class IV (partly skilled e.g. Postmen), social class V (unskilled e.g., porters, labourers), and non-employed VI (retired, unemployed, housewives). |
| Fekadu et al., (2010) | Education (illiterate or read/write only, elementary, secondary) |
| Gajalakshmi et al., (1997) | Education (Illiterate; <= 5 years, 6-12 years, > 12 years, Unknown) |
| Gavidia et al., (2012) | Parent education, household income, clean water at home, toilet at home |
| Gupta et al., (1994) | Subjects who had not received any formal education were placed in the uneducated group (group 1). Number of years of education was calculated from the highest class achieved in school or college. Educated subjects were subdivided in groups of five years: up to five years of education (group 2) corresponded to primary school level, up to 10 years (group 3) to secondary school level, and more than 10 years (group 4) to higher secondary or college level education. |
| Gupta et al., (2003) | Educational status was classified into Group 0: no formal education, Group I: 1-10 years, Group II: 11-15 years, and Group III: >16 years. |
| Gupta et al., (2012) | Socioeconomic status (monthly purchasing power parity, comparing the price of a standard ‘basket’ of goods and services), Parent education |
| Hoang et al., (2006) | Education and Household Economic Condition: Education level was classified as either no formal education (including illiteracy) or formal education (completion of any level of schooling). Economic condition of households was described as either poor (average monthly income per person less than VND 45,000 or U.S. $3.30) or non-poor (average monthly income per person more than or equal to VND 45,000 or U.S. $3.30) |
| Jerliu et al., (2013) | Educational level (0 years, 1–8 years and ≥9 years), self-perceived poverty level (dichotomized into: not poor vs. poor) |
| Jordan et al., (2013) | Property level (low, medium, high), based on five property parameters: radio, bicycle, television, motorbike, and car. The values of the objects available in the households were totalled to a ‘‘property index’’ which was grouped into three levels: low, medium, and high. |
| Khan et al., (2015) | Education status (<8 or > 8 school years); Socioeconomic Status (monthly income of PKR <15,000/month = poor/low, >15,000 month = middle/high) |
| Kinra et al., (2010) | Standard of living index from answers to a questionnaire, divided into 3 |
| Kisjanto et al., (2005) | SES (Low, Middle, High), based on information of income, type of housing and common luxury household items using previously published methods by Indonesian bureau of statistics |
| Lan et al., (2013) | Education level obtained from interviews with patients or their relatives. Five groups: illiterate, primary school, secondary school, high school and junior college, or higher. |
| Le Tran et al., (2001) | Education: Illiterate, finished primary or some of primary school, finished secondary school, finished upper secondary school or graduated vocational, college, university |
| Minh et al., (2003) | Education (I. less than high school, II: high school and above; Economic condition: I. Poor (less than 15 kg rice, D 45,000 (Vietnamese dong), or 3.3 USD)) |
| Misganaw et al., (2013) | Education (<High school, ≥ secondary school) |
| Miszkurka et al., (2012) | Education (no formal education, some formal education); Annual household income (based on self-reported expenditure on food, housing, health care costs and all other goods and services in the past four weeks, based on household annual consumption per capita to create four income categories 1. poorest (annual income less than one-half of poverty line, 2. below poverty line but about the poorest, 3. above but less than double the poverty line and 4) more than double the poverty line. |
| Mostert et al., (2012) | Assigned hospital class at diagnosis (based on parental income) or insurance status (3 types: 1. civil servants; 2: private-sector employees; 3: the poor). This was dichotomised into 'prosperous' or 'poor' |
| Pais et all, (1996) | Level of education: None, School, College; Level of income per month: <Rs 1999; Rs 2000-3999; >= Rs 4000 |
| Parkin et al., (2000) | Level of education (no school, primary, secondary, university) |
| Patil et al., (2014) | Family income (higher vs lower). Higher ≥ 5,000 Indian rupees per month, Lower <5,000 rupees per month |
| Pednekar et al., (2011) | Subjects were divided according to educational status into one of the five groups: illiterate, primary school (≦ 5 years of formal education), middle school (6-8 years), secondary school (9-10 years) and college (> 10 years) |
| Ploubidis et al., (2013) | Education (Primary, Secondary, College/University, None); Assets (building materials of the house-- types of walls, roof, floor and toilet; ownership of household assets-- radio, TV, fridge, phone, cupboard, sofa set, sewing machine, table, bicycle and vehicle, animals) |
| Rao et al., (2011) | Per capital household consumption (grouped into poorest 40%, middle 40% and richest 20%) using population distributions. |
| Rastogi et al., (2011) | Household income (Rupees per month: 3000, 3000–6000, 6000–10 000, >10 000) |
| Reddy et al., (2007) | Educational status (Four groups: 1 Postgraduate,2 Beyond secondary school, 3 above primary and up to secondary, 4 Uneducated/up to primary). More than one-fifth (22.6%) of the study group were uneducated or had education only up to primary school. The majority of the individuals (43.3%) had education above primary school and up to secondary school. A small proportion (12.9%) of the study group were postgraduates. The remaining 21.2% were either college graduates or had studied beyond the secondary-school level. |
| Rossier et al., (2014) | Household standard of living based on presence of durable goods (refrigerator and television) and most expensive mode of transport available in the home(e.g. bicycle, motorcycle, car). Derived coefficients for each good using Principal Component Analysis. Based on the scores, created three categories of household wealth (poorest, middle, wealthiest); Education (none, primary, secondary or more) |
| Safraj et al., (2012) | Socioeconomic status (1 low to 4 high), based on family assets, land holding, nature and quality of residence (toilets, safe water, electricity), ownership of animals and commonly used consumer electronic items (e.g. radio, television, telephone, camera, washing machine, microwave, and other kitchen equipment. Ownership of transportation vehicles.). Participants were given a household score which was divided into 4. |
| Samuel et al (2012) | Individual’s education level, paternal education and a score based on household possessions as indicators of SES. Education variables were recorded as one of four categories from ‘no schooling’ (category 1, 0 years), primary and middle school (category 2, 1–8 years), high school and higher secondary (category 3, 9–12 years) and 412 years of schooling (category 4,412 years). Paternal education was used as a measure of childhood SES. For the possessions score, participants were asked if the household owned each of the following items/ amenities: electricity, fan, bicycle, radio, motorized two-wheeled vehicle, gas stove, television, cable television, electric mixer, electric grinder, electric air cooler, washing machine, car, air conditioner, computer, television antenna and telephone. They created a composite score using weights obtained from principal component analysis (PCA) and grouped the first principal component by quintiles. |
| Sankaranarayanan et al., (1995) | SES assessed by social workers employed by hospital (based on annual income, occupation, ownership and type of house, ownership of household items such as television, refrigerators, etc., vehicles and land properties and agricultural income. Low SES (monthly income < 500 Rs, high SES (monthly income > 3000 RS, ownership of land, house, car, etc; Education (Nil, Primary, Upper primary, High school and college, not known) |
| Sayeed et al., (1997) | Rural poor (landless farmers subsisting on agrarian labor); rural rich (landholders) |
| Sharieff et al., (2003) | Socioeconomic class (authors did not describe how it was determined) |
| Singh et al., (1997) | SES scale (1-4), based on Raman Kutty classification which looks at education, occupation, housing conditions, ownership of consumer durables, ownership of land, per capita total income. 1 was highest and 4 was lowest. |
| Singh et al., (2005) | SES based on attributes of housing conditions, education, occupation, per capita income and ownership of consumable durables like a car. |
| Van Minh et al., (2008) | Education (less than secondary school, secondary school, high school and higher); Economic condition of household (poor: average income per person per month less than VND 100,000 or US $6.3); middle (average income per person per month 100,000-600,000 or US $6.3-63) and rich (average income per person per month is greater than VND 600,000 or US $63.0 |
| Xavier et al., (2008) | SES: Rich, Upper middle class, Lower middle class and Poor based on physician record of education, occupation, family income and properties such as houses, cars and land. |
| Xin et al., (2014) | Education level (Illiterate and primary school vs middle school and higher) |
| Yadav Ret al., (2013) | Occupation (service (salaried), business and "others", which mainly included people who were unemployed or who were daily wage workers, unskilled labourers or farmers without their own land). |
| Zaman et al., (2012) | Information on socio-economic variables was collected by self-report. For each participant the highest level of education completed was recorded from the categories: higher education (diploma/technical/ university); secondary school (to grade 12); primary school (to grade 6) or no formal schooling. They defined ‘Educated’ as those who had attended primary school, secondary school or higher education and ‘No education’ as those reporting no formal schooling. Household income was recorded in Indian rupees for an average month. For seasonal workers, interviewers were trained to assist in calculating an average monthly income based on the last 12 months income. |
