# Supplementary material for: A systematic review of associations between non-communicable diseases and socioeconomic status within low- and lower-middle-income countries
Source: J Glob Health. 2018 Jul 25;8(2):020409. doi: 10.7189/jogh.08.020409 (PMC6076564; doi:10.7189/jogh.08.020409)
Supplement: Online Supplementary Document [file jogh-08-020409-s001.zip › Appendix_4.1_Cancer_All_Results_v3.pdf]

Appendix 4.1 Full results among papers reporting cancer outcomes

Study	Quality	Outcome		Exposure		n	Crude	%	95% CI	p	Adjusted		95% CI	p
Ali et al. (2008)	High	Late stage diagnosis of breast cancer (stages III and IV)				n		%				OR		
				SES	Lowest SES	190		41.5				1	ref	0.21
					Medium SES	131		24				0.67	0.41-1.12	
					Highest	201		34.5				1	0.59-1.72	
				Education	Illiterate	46		14.5				2.72	1.06-7.03	0.045
					Literate/Primary	75		28.4				2.32	1.05-5.13	
					Middle	126		26.5				2.07	1.02-4.21	
					Secondary	167		28.4				1.16	0.63-2.16	
					> College	99		14.5				1	ref	
					Unknown	9		1.8				n/a		
										Adjustments: age, district, religion, marital status, income, education, occupation				
Aziz et al. (2008)	High	Stage I-III breast cancer	Event-free survival	SES	Poor SES	171		32.6				1	ref	
					Middle/High	354		67.4				0.6	0.45-0.8	<0.0001
			Overall survival											
					Poor SES	171		32.6				1	ref	
					Middle/High	354		67.4				0.6	0.41-0.88	0.009
Adjusted models control for age, tumour size, grade of tumour, nodal status, hormone receptor status, initial stage at diagnosis, adjuvant radiation, chemotherapy														
Aziz et al. (2010)	High	Breast cancer (locally advanced)	Event-free survival	SES	Low/Middle SES	202		85.2						0.05
					High SES	35		14.8						
			Overall survival		Low/Middle SES									0.1
					High SES									
Aziz et al. (2004)	High	Breast Cancer	Distribution of participants	SES	Low SES	99		35						
					Intermediate SES	126		44						
					High SES	61		21						
			Size of tumor (71)		Low SES	2		2		<.001				
					Intermediate SES	10		7.9						
					High SES	12		19.6						
			Size of tumor (72)		Low SES	38		38.3						
					Intermediate SES	67		53.1						
					High SES	34		55.7						
			Size of tumor (73)		Low SES	20		20.2						
					Intermediate SES	24		19						
					High SES	10		16.3						
			Size of tumor (74)		Low SES	20		20						
					Intermediate SES	24		19						
					High SES	5		8.1						
			Stage at diagnosis (I)		Low SES	1		1		<.001				
					Intermediate SES	5		3.9						
					High SES	10		16.3						
			Stage at diagnosis (II)		Low SES	40		40.4						
					Intermediate SES	72		57.1						
					High SES	33		54						
			Stage at diagnosis (III)		Low SES	39		39.3						
					Intermediate SES	38		30.1						
					High SES	14		22.9						
			Stage at diagnosis (IV)		Low SES	19		19.9						
					Intermediate SES	11		8.7						
					High SES	4		6.5						
			Mean number of lymph nodes positive		Low SES			3		194				
					Intermediate SES			4						
					High SES			2						
			Mean time elapsed before diagnosis (months)		Low SES			10.6	8.1-13.1	<.001				
					Intermediate SES			6.4	4.8-7.9					
					High SES			4.31	2.5-6					
			Mean age at diagnosis (years)		Low SES			43	41-45	<.001				
					Intermediate SES			46	44-48					
					High SES			50	47-53					
			5 year disease free survival		Low SES			0.31		<0.0001				
					Intermediate (Not Available)			0.48						
					High SES			0.79						
			5 year overall survival		Low SES			0.49		<.0001				
					Intermediate (Not Available)			0.68						
					High SES			0.86						

Bonilla et al., (2010)	High	Acute Lymphoblastic Leukemia			Parental Education									
					**Standard Risk**	n		%				HR	95% CI	p
			Event-free survival		Illiterate	8		0.031				Parental secondary education		
					Primary	167		0.652				0.49	(0.29, 0.84)	0.01
					Secondary	56		0.219				Monthly income (per $100)		
					Advanced	22		0.086				0.84	(0.70, 0.99)	0.04
					**High Risk**									
					Illiterate	8		0.044				Parental secondary education		
					Primary	111		0.613				0.69	(0.42, 1.14)	0.15
					Secondary	43		0.238				Monthly income (per $100)		
					Advanced	18		0.099				0.91	(0.80, 1.03)	0.14
Adjusted models control for age, sex, disease related features (WBC count, uric acd, DNA index, etc.), monthly income, time of travel to clinic, parental income, presence of phone, mode of transport, BMI (and TSF percentile, mid upper arm circumference)														
Chankapa et al., (2011)	High	Cervical Cancer												
			Low-grade squamous intraepithelial lesion	Education	Educational Status	n (overall)	n (outcome)	%		Comparing literate to illiterate (Chi-squared with Yate's correction)				
					Illiterate	16	5	10.7		p = 0.1				
					≤ Class V	15	2	4.2						
					≤ Class Viii	7	-							
					≤ Class X	8	-							
					≤ Class Xii	1	-							
					Total (%)	47	7	14.8						
				Income	Per Capita Monthly Family Income (Rs)									
					≤ 500	35	7	14.8						
					501-1000	7	-	-						
					1001-1500	3	-							
					1501-2000	1	-							
					2001-2500	1	-							
			High-grade squamous intraepithelial lesion		Total (%)	47	14	29.7						
				Education	Educational Status		1	2.1						
					Illiterate		-							
					≤ Class V		-							
					≤ Class Viii		-							
					≤ Class X		-							
					≤ Class Xii		1	2.1						
					Total (%)					Comparing PCMFI ≤ 500 Rs to >500 Rs)				
				Income	Per Capita Monthly Family Income (Rs)					0.6291				
					≤ 500		35	74.5						
					501-1000		7	14.8						
					1001-1500		3	6.4						
					1501-2000		1	2.1						
					2001-2500		1	2.1						
					Total (%)		47	100						
Chaouki et al., (1998)	High	Cervical Cancer		Education			Cases		Controls					
							n	%	n	%		OR	95% CI	
					None		188	87.9	116	57.1		1		0.0001
					Primary		18	8.4	29	14.3		0.52	(0.27–1.01)	
					Secondary		6	2.8	34	16.7		0.17	(0.07–0.42)	
					Higher		2	0.9	24	11.8		0.08	(0.02–0.34)	
				Facilities at home										
												OR	95% CI	
					>4		35	16.4	101	49.8		1		0.0001
					3–4		85	39.7	68	33.5		3.36	(1.98–5.70)	
					1–2		48	22.4	27	13.3		5.62	(2.95–10.69)	
					None		46	21.5	7	3.4		14.59	(5.86–36.32)	
				Family Income (Cases)										
												OR	95% CI	
					High		15	7.1	78	38.4		1		0.0001
					Middle		56	26.5	59	29.1		3.98	(2.01–7.90)	
					Low		140	66.4	66	32.5		8.17	(4.28–15.57)	
					Unknown		3		0			8.17	(4.28–15.57)	
Dutta et al., (2005)	High	Age Of Diagnosis For Gallbladder Cancer			Lower SES		OR for younger gallbladder diagnosis and lower SES					OR	95% CI	p
					Young (≤50 Years) Gallbladder Cancer (N=51)	45						3.1	1.1-8.6	0.03
					Older (>50 Years) Gallbladder Cancer (N=70)	50								
											Adjusting for age, sex , residence, rural origin, smoking, gallstones			
Gajalakshmi et al., (1997)	High	Breast Cancer Survival Rate (5 year)			Education	n	%					HR	95% CI	p
					Illiterate	571	46.8				Illiterate	1	ref	0.05
					<5 Years	364	51.1				<5 Years	0.92	(0.75-1.13)	
					6-12 Years	562	46.1				6-12 Years	0.99	(0.82-1.20)	
					>12 Years	124	69.5				>12 Years	0.54	(0.37-0.80)	
					Unknown	126	25.7				Unknown	2.57	(1.89-3.48)	
							<0.001				*Adjusting for age, marital status, education, extent of disease*			

Gavidia et al., (2012)	High	Sepsis And Infectious Deaths Among Leukemia Patients	Mother's education	Mother's Education (N=230)	n	%				Sepsis (n=47)	OR		
				Advanced	32	13.9				Mother illiterate	3.06	1.09, 8.63	0.034
				High School	39	17				Father illiterate	0.54	0.13, 2.18	0.384
				Secondary School	48	20.9				Annual Household Income <$2000	1.02	0.45, 2.31	0.966
				Primary School	83	36.1				No clean water at home	0.69	0.34, 1.43	0.318
				Illiterate	28	12.2				No toilet at home	1	0.50, 1.98	0.992
			Father's education	Father's Education (N=175)									
				Advanced	22	12.6				Infection Death (n=7)			
				High School	26	14.9				Mother illiterate	3.63	0.73, 17.95	0.114
				Secondary School	40	22.9				Father illiterate	2.26	0.24, 21.72	0.48
				Primary School	70	40				Annual Household Income <$2000	13.9	1.62, 119.10	0.016
				Illiterate	17	9.7				No clean water at home	1.76	0.38, 8.12	0.47
										No toilet at home	2.05	0.38, 10.95	0.402
			Household Characteristics	Household Characteristics									
				Annual Household Income <$2000 Us(%)	89	35.5							
				No Clean Water At Home (%)	108	43							
				No Toilet At Home (%)	128	51				*Adjusting for age, sex, type of cancer, characteristics of episode onset.*			
Gupta et al., (2012)	High	Leukemia (Treatment related mortality)								**Predictors of early death**	OR	95% CI	p
										Monthly purchasing power parity u	1.11	0.99, 1.25	0.07
										Parental education, at least second	0.45	0.12, 1.64	0.23
										Adjusting for sex, age, clinical and nutritional features			
Jordan et al., (2013)	High	Breast Cancer	Property level	Cases	Controls					**Property level**	OR	95% CI	p
				115	230					Low	Ref		
										Medium	0.34	0.19-0.61	<.05
										High	0.22	0.09-0.55	<.05
										Adjusting for age, place of living			
Khan et al., (2015)	Medium	Presentation delay for breast cancer	SES		n	%					OR	95% CI	p
				Poor/Low	269	85.40				Education (< 8 years)	2.26	1.25-4.10	<.05
				Middle/High	46	14.60				SES (low to middle)	2.29	1.06-4.94	<.05
			Education	<8 Years Of School	234	74.29							
				>8 Years Of School	81	25.71							
Lan et al., (2013)	High	Breast Cancer (Survival Probability)	Education		n						HR	95% CI	p
				Illiterate	40					Illiterate	1	baseline	-
				Primary School	285					Primary school	1	0.83-1.20	0.974
				Secondary School	264					Secondary school	0.95	0.78-1.14	0.571
				High School	250					High school	0.79	0.64-0.96	0.02
				Higher	109					Higher	0.79	0.62-0.99	0.046
										Extended cox model assessing effect:			
										Education level	0.92	0.89-0.95	<.05
										Adjusting for marital status, hormone therapy, stage at diagnosis,			
Misganaw et al., (2013)	High	Malignant Neoplasm		Educational Status Of Sample (N=3709)	n	%	Association of Education with malignant neoplasm mortality (n=386) in Addis Ababa						
			Males	No Education	484	26		Crude OR			Adjusted OR	95% CI	p
				Elementary School	486	26	< high school	Ref		< high school	ref		
				Secondary School	465	25	≥ secondary school	1.12	(0.89-1.41)	≥ secondary school	1.49	1.12-1.99	0.006
				College/University	222	12				*Adjusting for sex, religion, marital status, ethnicity, occupation*			
				Other (Church Education)	207	11							
			Females	No Education	971	53							
				Elementary School	405	22							
				Secondary School	276	15							
				College/University	74	4							
				Other (Church Education)	119	6							
			Among those with malignant neoplasms										
				< High School	270	0.07							
				≥ Secondary School	116	0.03							
Mostert et al., (2012)	High	Childhood cancers	Poor (n=112)			%					OR	95% CI	p
				Treatment abandonment		54				Prosperous	ref		
				Death		36				Poor	3.3	1.4-8.1	0.006
				Progressive or relapsed disease		7							
				Event-free survival		4							
			Prosperous (n=31)										
				Treatment abandonment		26							
				Death		29							
				Progressive or relapsed disease		16							
				Event-free survival		29							
Parkin et al., (2000)	High	Non-Hodgkin Lymphoma	Education		Cases (n)	Controls (n)	OR	95% CI			OR (Adjusted)	95% CI	p
				No School	3	34	1	ref			1		
				Primary	14	68	2.4	(0.6±10.8)			2.1	(0.4 ± 11.1)	
				Secondary	17	42	4.4	(1.0±20.2)			3.5	(0.6 ± 20.5)	
				University	6	11	7.4	(1.3±42.2	0.009		7.9	(0.9 ± 73.3)	0.09
Adjusted model controls for HIV, Residence, Education, Occupation, Travel, History of STD													

Patil et al., (2014)	High	Hepato Cellular Carcinoma	Family income (Cases)		Cases (n)	Controls (n)		OR	95% CI	p		OR	95% CI	p
				Higher	92	138		1.39	0.90-2.13	0.1361		1.9	1.07-3.36	0.0276
				Lower	49	102								
Sankaranarayanan et al., (1995)	High	Cervical Cancer (5 year survival)	Education		n	%	5 year survival	p-val				HR	95% CI	p
				Nil	172	38	45.2	0.93			Low	1	reference	<0.05
				Primary school	139	31	49.4				Middle	0.7	0.5-1.1	
				Upper primary school	80	18	50.8				High	0.2	0.04-0.8	
				High school and college	46	10	43.9				Not known	2.1	0.2-15.4	
				Not known	15	3	45.4				*Adjusts for stage of disease, performance status*			
			SES											
				Low	345	76	43.6	<0.05						
				Middle	85	18.7	54.6							
				High	18	4	85.6							
				Not known	4	1	50							
Xin et al., (2014)	High	Colorectal cancer	Education level									OR	95% CI	p
											Illiterate and primary school	1		
											Middle school and higher	2.39	1.76-3.25	<.001
Le Tran et al., (2001)	Medium	Cervical Cancer	Education		n	%						RR	95% CI	
				Finished upper secondary school or graduated vocational, university	252	5					Finished upper secondary school or graduated vocational, university	1	reference	
				Finished secondary school	508	10.1					Finished secondary school	0.83,	0.68-1.00	
				Finished primary or some of primary school	2764	54.9					Finished primary or some of primary school	1.53,	1.30-1.81	
				Illiteracy	1510	30					Illiteracy	3.43	2.85-4.14	<.05
