# Supplementary material for: A systematic review of associations between non-communicable diseases and socioeconomic status within low- and lower-middle-income countries
Source: J Glob Health. 2018 Jul 25;8(2):020409. doi: 10.7189/jogh.08.020409 (PMC6076564; doi:10.7189/jogh.08.020409)
Supplement: Online Supplementary Document [file jogh-08-020409-s001.zip › Appendix_4.2_CVD_All_Resultsv2.pdf]

Appendix 4.2 Full results among papers reporting CVD outcomes

Study	Outcome	Quality	Outcome	Exposure		n		Crude	%	95% CI	p	Adjusted		95% CI	p
Das et al., (2007)	Age-standardised prevalence and i	High	Stroke	Education (slums)		Slums	Non-slums					**Slums**			
						n	n					Prevalent cases of stroke	57		
					Primary (Standard I To X)	7,268	4,207					Prevalence rate per 100,000 persons	517.95	395.19–667.1	NS (p>0.05)
					Higher (Standard Xi And Higher)	1,651	22,135					Incident cases of stroke	29		
				Income (per family per month) (slums)								Average annual incidence rates per 100,000	131.76	88.94–188.15	NS (p>0.05)
					Rs 2500 (Us$ 56)	252	1,232					Case fatality rate	9		
					Rs 2500–5000 (Us$ 56–111)	1,850	5,729						0.3103	14.25-58.89	NS (p>0.05)
					Rs 5000 (Us$ 111)	219	2,537								
												**Non-slums**			
												Prevalent cases of stroke	190		
												Prevalence rate per 100,000 persons	459.24	396.32–530.72)	
												Incident cases of stroke	100		
												Average annual incidence rates per 100,000	120.85	98.37–146.95	
												Case fatality rate	44		
													0.44	31.77-59.18	
												Note: p values above are for comparison between slum and non-slum prevalence rates (age standardized)			
Dogra et al., (2012)	AMI (WHO criteria)	High	AMI	Low SES		n		OR (Comparison among patients and controls, X2)					OR (Multiple logistic-regression adjusting for age and sex)		
					Controls	86			1.9	1.3-2.8	<0.001		13.5	2.3-78.4	0.004
					Cases	71							*Adjustments: age and sex*		
Engels et al., (2014)	Stroke (diagnosed following WHO	High	Stroke	Wealth (Overall)									Coeff	95% CI	p
					Rich								1		
					Intermediate								1.35	(0.84;2.17)	0.212
					Poor								1.47	(0.92;2.32)	0.104
				Wealth (rural)		16,563									
					Rich								1		
					Intermediate								1.06	(0.56;1.99)	0.86
					Poor								0.54	(0.26;1.13)	0.104
				Wealth (urban)		28,179									
					Rich								1		
					Intermediate								1.84	(0.98;3.44)	0.057*
					Poor								2.06	(1.09;3.89)	0.025**
Adjustments: Age, Sex, hypertension, diabetes, heart diseases, neuropsychiatric disorders, other diseases. In models, dropped non-sig factors: education, occupation, tobacco smoking, practice of sport, respiratory disorders															
Enkh-Oyun et al., (2013)	IHD using the Rose questionnaire a	High	IHD	Education					Unadjusted				Adjusted		
				Overall					OR	95% CI			OR	95% CI	
					Elementary	230			1	X			1	X	
					Incomplete Secondary	531			0.87	(0.59–1.28)			0.89	(0.59–1.35)	
					Complete Secondary	606			0.79	(0.54–1.16)			0.88	(0.58–1.37)	
					Vocational	357			0.68	(0.44–1.05)			0.75	(0.44–1.27)	
					University	556			0.56	(0.37–0.84)*			0.64	(0.38–1.09)	
				Men	Elementary	68			1	X			1	X	
					Incomplete Secondary	253			1.36	(0.65–2.85)			1.58	(0.72–3.47)	
					Complete Secondary	221			1.24	(0.58–2.64)			1.61	(0.71–3.67)	
					Vocational	82			0.72	(0.27–1.88)			1.04	(0.35–3.07)	
					University	227			0.82	(0.37–1.78)			1.3	(0.49–3.50)	
				Women	Elementary	162			1	X			1	X	
					Incomplete Secondary	278			0.72	(0.45–1.17)			0.75	(0.45–1.27)	
					Complete Secondary	385			0.66	(0.42–1.04)			0.7	(0.41–1.19)	
					Vocational	275			0.64	(0.39–1.05)			0.59	(0.31–1.10)	
					University	329			0.49	(0.30–0.81)*			0.5	(0.26–0.97)*	
Hoang et al., (2006)	CVD mortality	High	Cardiovascular Disease Mortality	Education (Men)	Men	n	%						Risk Ratio	95% CI	
					No Formal Education	699	4.9					Formal education	ref		
					Formal Education (Having Attended Any I	13,590	95.1					No formal education	4.5	3.4-5.8	
				SES (Men)	Poor (Monthly Income < Vnd 45,000, Us	1,624	11.8					Poor	ref		
					Non-Poor (Monthly Income > Vnd 45,00(	12,174	88.2					Non-poor	1.1	0.8-1.6	
				Education (Women)	Women										
					No Formal Education	3,012	18								
					Formal Education (Having Attended Any I	13,701	82								
				SES (Women)	Poor	2,278	14.1								
					Non-Poor	13,871	85.9								
				Education (Overall)	Overall										
					No Formal Education	3,711	12								
					Formal Education (Having Attended Any I	27,291	88								
				SES (Overall)	Poor	3,902	13								
					Non-Poor	26,045	87								

Kisjanto et al., (2005)	Stroke (first time stroke, based on	Medium	Stroke	Social class		Controls		Controls							
				(Controls)	Social Class	n	%	n	%				OR	95% CI	p
					Lower Class	59	25.1	151	22.1			Lower class	ref		
					Middle Class	104	44.3	266	39			Middle class	0.95	0.62-1.45	0.81
					Higher Class	72	30.6	265	38.9			Higher class	0.65	0.41-1.03	0.06
				Education (control)	Secondary Or Higher Education	132	56.2	434	63.6			Secondary or higher education	0.72	0.52-0.99	0.04
												Adjusting for medical history, age, being single			
Minh et al., (2003)	CVD death (determined using WH(	High	CVD Death (Determined Using Who	Education		Mortality rate per 1,000 person years (cases)			Univariate rate				Multivariate rate		
					High School And Higher	3	4		1			High school and higher	1		
					Less Than High School	6	87		2.55	0.94-6.94	NS	Less than high school	1	0.32-3.13	NS
				Economic Condition								Economic condition			
					Poor	5	9		1			Poor	1		
					Non-Poor	6	77		1.28	0.64-2.56	NS	Non-poor	1	0.69-2.74	NS
Miszkurka et al., (2012)	Angina	High	Angina					Crude prevalence					Adjusted prevalence ratio (PR)		
				Education				Prev (%)	95% CI				PR	95% CI	p
					No Formal Education			18.6	16.3-21.1			No Formal Education	ref		
					Some Formal Education			14.6	10.8-19.4	0.1037	0.1037	Some Formal Education	1.13	0.80-1.59	NS
				Annual Household Income											
				<PL/2	Less than half of poverty line			16.8	14.1-19.9			Less than half of poverty line	ref		
				≥PL/2-<PL	Below the PL but above the poorest			19.7	16.3-23.5			Below the PL but above the poorest	1.24	1.01-1.53	p<0.05
				≥ PL-< 2 X PL	Above the poverty line but less than double the poverty line			17.7	14.5-21.4			Above the poverty line but less than double	1.21	0.96-1.52	
				≥ 2 X PL	More than double the poverty line			16.6	13.2-20.7	0.3961	0.3961	More than double the poverty line	1.25	0.93-1.68	
												Adjusted for gender, age, setting, marital status.			
Sharieff et al., (2003)	6 month outcome (recovery---grou	High	6 Month Outcome From Peripartum Cardiomyopathy				Group A (Imp	n	%						
			Recovery	SES	Fair	8	Fair	6	50%						
					Poor	27	Poor	6	50%						
							Group B (Deteriorated n=23)								
			Deterioratior	SES	Fair		Fair	2	8.69						
					Poor		Poor	21	9.13		p=0.011			Poor SES**	p=0.011
	NB: P value is for comparison and correlation with Pearson's correlation coefficient and student's t test (comparing SES fair or poor between Group A (improved condition) and group B (deteriorated condition)														
Singh et al., (1997)	CAD (angina, Rose questionnaire, E	High	CAD									Social class and prevalence of CAD			
				Social Class: Males (n-894)				n	%	MH Chi-squared			OR	95% CI	
					Social Class 1 (More Affluent)	147		17	11.5			Males	0.83	0.66-0.95	<.01
					Social Class 2	147		15	10.2			Females	0.61	0.42-0.81	<.05
					Social Class 3	287		2	0.7						
					Social Class 4 (Less Affluent)	313		1	0.3			Males	0.96	0.82-1.18	NS
					Total			35	3.9	12.6	<0001	Females	0.81	0.50-1.09	NX
				Social Class: Females (n-875)											
					Social Class 1	115		12	10.4						
					Social Class 2	112		9	8						
					Social Class 3	313		2	0.6						
					Social Class 4	335		--							
					Total			23	2.6	9.5	<0001				
Model 1 adjusted for blood pressure, cholesterol, diabetes, physical activity, smoking.															
Model 2 adjusted for smoking, sedentary lifestyle, BMI, blood pressure and cholesterol															
Xavier et al., (2008)	Mortality within 30 days	High	Mortality Within 30 Days Following Acute Coronary Syndromes					30 day mortality rates (Crude)				30 day mortality rates (Adjusted)			
				SES		**n**						**Model 1**	OR	95% CI	
					Rich	1,078		Rich	5.5			Rich	ref		
					Upper Middle Class	4,590		Upper middle class	5.9			Upper middle class	1·11	(0·79–1·56)	
					Lower Middle Class	10,737		Lower middle class	6.5			Lower middle class	1·25	(0·91–1·71)	
					Poor	3,999		Poor	8.2			Poor	1·44	(1·03–2·02)	0·04
												**Model 2**			
												Rich	ref		
												Upper middle class	0·95	(0·47–1·90)	
												Lower middle class	0·88	(0·45–1·72)	
Model 1 adjusted for risk factors and location of infarct												Poor	0·90	(0·43–1·88)	0·97
Model 2 adjusted for risk factors, location of infarct and treatments (type of hospital, time to hospital, time to thrombolysis, drugs in hospital and interventional procedures)															

Yadav Ret al., (2013)	Morbidity (Barthel's index and mo	High	Post-stroke morbidity or mortality		SES				n	%						
						Upper Class			9	2.5						
						Middle Class			216	59.3						
						Lower Class			139	38.1		<0.0005				
					Education											
						Uneducated			98	16.4						
						Undergraduate			294	49.4						
						Graduate			192	32.2		<0.0005				
					Job status										Barthel Index (NB: See paper for more details)	
						1 Salaried			109	0.18381113				1 Salaried	16	
						2 Business			49	0.08263069				2 Business	8	
						3 Others			435	0.73355818				3 Others	52	
														Total	76 (12.7%)	0.018
Gupta et al., (1994)	Prevalence of coronary heart disea	High	CHD						n	%						
				Education												
					Men	Group 1 (Nil)			36	4.7						
						Group 2 (1.5 Years)			8	2.3						
						Group 3 (6-10 Years)*			14	2.4						
						Group 4 (>10 Years)			10	3.6		0.093	no			
					Women	Group 1 (Nil)			32	5.3						
						Group 2 (1.5 Years)			6	2						
						Group 3 (6-10 Years)*			5	2		0.007	yes			
						Group 4 (>10 Years)			-							
Pednekar et al., (2011)	Cardiovascular mortality	High	CVD		Education					Crude				Age-adjusted		
					(Men)		Age-adjusted death rates per 100,000			HR	95% CI			HR	95% CI	p
						Illiterate	471		Illiterate	1				1		
						Primary School	654		Primary school	1.33	(1.20-1.48)			1.36	(1.22-1.51)	
						Middle School	618		Middle school	0.8	(0.71-0.90)			1.27	(1.13-1.43)	
						Secondary School	518		Secondary school	0.62	(0.52-0.73)			1.01	(0.85-1.19)	
						College	450		College	0.71	(0.59-0.85)			0.88	(0.73-1.06)	<.05
					Education				Women							
					(Women)	Illiterate	429		Illiterate	1				1		
						Primary School	301		Primary school	0.41	(0.35-0.47)			0.69	(0.60-0.80)	
						Middle School	267		Middle school	0.17	(0.12-0.23)			0.55	(0.40-0.76)	
						Secondary School	426		Secondary school	0.34	(0.23-0.51)			1.04	(0.69-1.57)	
						College	317		College	0.26	(0.13-0.56)			0.74	(0.35-1.56)	NS
Rastogi et al., (2011)	Acute myocardial infarction	High	AMI		Education		Cases (n)	Controls (n)		Age- sex-adjusted RR				Multivariate RR		
					(Cases)		N									
						Highest level	17	59		1				1		
						None	42	84		2.1	(1.1,4.1)	<.05		2.5	(1.0,6.0)	<.05
					Household income											
					(Cases)	>10000 Rupees/Month	69	161		1				1		
						<3000 Rupees/Month	107	161		1.6	(1.1,2.5)	<.05		1.6	(0.9,3.0)	NS
Adjusted models control for age, sex, hospital, cigarette smoking, BMI, waist-to-hip ratio, physical activity, history of hypertension, diabetes, high cholesterol, family history of IHD, alcohol intake, education, household income and Hindu religion.																
Pais et all, (1996)	Acute myocardial infarction	High	AMI		Income (Cases)		Cases (n)	Controls (n)						Adjusted OR	95% CI	
						<Rs 1999	91	74					Income <4000 Rs/month	2.67	1.20, 5.94	
						Rs_2000–3999	81	78								
						≥ Rs 4000	28	48								
					Education (Cases)											
						None	17	21								
						School	118	95								
						College	65	84								
												Controlling for smoking, waist hip ratio, hypertension, fasting blood glucose, non-vegetarian				
