# Supplementary material for: A systematic review of associations between non-communicable diseases and socioeconomic status within low- and lower-middle-income countries
Source: J Glob Health. 2018 Jul 25;8(2):020409. doi: 10.7189/jogh.08.020409 (PMC6076564; doi:10.7189/jogh.08.020409)
Supplement: Online Supplementary Document [file jogh-08-020409-s001.zip › Appendix_4.3_Diabetes_Full_Resultsv2.pdf]

Appendix 4.3 Full results among papers reporting Diabetes Outcomes

Study	Quality	Outcome	Exposure				Crude	%		Adjusted	95% CI	p
Bella et al. (1992)	High	Insulin Dependent Diabetics	SES				Diabetics	Non-Diabetics				
							%	%				
				Upper Social Class			9.3	10	<.001			
				Middle Social Class			41.9	38	<.001			
				Low Social Class			48.8	52	<.001			
			Education									
				Completed Primary Education			74.4	100	<.001			
				Completed Secondary Education			18.6	60	<.001			
				Attending Polytechnic/University			4.7	23	<.001			
				Graduated From Polytechnic/University			2.3	6	>.1			
			Employment status									
				Nonskilled			46.5	40	>.1			
				Skilled			18.6	50	<.001			
				Unemployed			34.9	10	<.001			
				NB: Comparing diabetics to non-diabetics								
Fekadu et al., (2010)	Medium	Insulin-Requiring Diabetes			Cases (n=107)		Controls (n=110)			OR	95% CI	
				Occupation	n	%	n	%				
				Farmer/Unskilled	53	55	27	28		3.5	(1.5–7.8)	<.001
				Housewife	9	9	10	11		1.6	(0.5–5.7)	
				Student	19	20	30	32		1	(0.4–2.6)	
				Government/Business	15	16	28	30		1		
				Not known/Unemployed	11	15						
				Education								
				Illiterate/Read/Write	46	43	22	20		4	(2.0–8.0)	<.001
				Elementary	28	26	26	24		2	(1.0–4.0)	
				Secondary/Higher education	33	31	62	56		1		
				Household Amenities								
				Animals Sleep In The Same Roor	20	19	16	15		1	(0.4–2.0)	0.9
				No Access To Toilet	26	24	11	10		3.6	(1.5–8.8)	0.004
				No Access To Piped/Clean Wate	44	41	28	26		2.3	(1.2–4.1)	0.009
				Hatched (Vs Corrugated) Roof	35	33	24	22		1.8	(0.9–3.3)	0.075
				No. Of People/House	4.9	2.2	4.6	2.2		0.3	(0.3–0.9)	0.28
				No. Of People/Room	2.4	1.9	2	1.9		0.4	(0.02–0.9)	0.04
				Distance From Clinic, Kmb	6.6	3.2	4.1	3.8		2.5	(0.6–5.2)	0.01
				Possessions Index	3.4	1.4	3.9	1.5		0.5	(0.1–0.9)	0.01
										Adjusted for: interviewer, location, gender and age		

Ploubidis et al., (2013)	High	Diabetes (Diagnosed By Glucose Reading, Doesn't Specify Type I Or Type Ii)	Material Resources (rural)		2959					0.562	0.404, 0.719	<.05
			Material Resources (urban)		1437					0.313	0.185, 0.431	
		NB: This paper used structural equation modelling to assess both direct and indirect effects of education via accumulated wealth and health behavior on prevalence of diabetes. Please see paper for more details. A										
								Adjusted for: age, sex, ethnicity and education				
Safraj et al., (2012)	Medium	Self-Reported Diabetes*	Socioeconomic Status	SES Group	n	diabetic (n)	percentage preval	95% CI	**SES Group 1 (poores**	Group 1 (n)	%	
				1 (Poorest)	9502	291	3.1	2.7-3.4	35-44 years	52	2.3	
				2	23344	1112	4.8	4.5-5.0	45-54 years	63	4.4	
				3	32420	2771	8.5	8.2-8.9	55-64 years	57	6.4	
				4 (Richest)	8494	946	11.1	10.5-11.8	65 years and older	88	8.6	
									**SES group 2**	Group 2 (n)	%	
									35-44 years	158	3.1	
									45-54 years	274	7.7	
									55-64 years	268	10.3	
									65 years and older	343	12.3	
									**SES group 3**	Group 3 (n)	%	
									35-44 years	338	5.2	
									45-54 years	711	13.2	
									55-64 years	759	19.1	
									65 years and older	849	20.9	
									**SES group 4 (wealthi**	Group 4 (n)	%	
									35-44 years	110	6.2	
									45-54 years	243	16.6	
									55-64 years	296	27.5	
									65 years and older	274	27.4	
									**Chi squared for difference between SES**		Chi-Square for tre	p value
									35-44 years		68.5	<.01
									45-54 years		175	<.01
									55-64 years		248.5	<.01
Diagnosed By Doctor On Basis Of Clinical And Biochemical Parameters									65 years and older		226.7	<.01
Reddy et al., (2007)	Medium	Diabetes	Education		n		Prevalence			Prev ratio	95% CI	p value for trend
			(Men)	Est I (Higher Education)	1611		8.4			1		
				Est II	2607		10.4			1.2	(0.9–1.6)	
				Est III	5820		13.3			1.6	(1.3–2.1)	
				Est IV (Lower Education)	1859		7.6			0.9	(0.6–1.4)	
				Total	11897							0.08
			(Women)									
				Est I (Higher Education)	960		4.2			1		
				Est II	1635		4.8			1.1	(0.6–1.8)	
				Est III	2832		9.8			2.4	(1.5–3.8)	
				Est IV (Lower Education)	2645		11.2			2.8	(1.8–4.4)	
				Total	8072							0.01
					19969				Adjusted for age and sex			

Corsi et al., (2008)	High	Self-Reported Diabetes Status			Models 1–3, OR (95% CI)				Model 5, OR (95% CI)		
			Social caste		CRUDE				Adjusted		
				Other Caste	1				1		
				Scheduled Caste	0.81	0.71, 0.94,			1.07	0.93, 1.24,	
				Scheduled Tribe	0.57	0.46, 0.70,			0.73	0.57, 0.92,	
				Other Backward Caste	0.84	0.75, 0.94,			0.96	0.86, 1.08,	
				No Caste	0.89	0.71, 1.11,			0.95	0.76, 1.20,	NS
			Wealth								
				Poorest	1				1		
				2Nd Quintile	1.59	1.20, 2.12,			1.49	1.14, 1.96,	
				3Rd Quintile	1.63	1.23, 2.16,			1.39	1.07, 1.81,	
				4Th Quintile	2.42	1.85, 3.17,			1.79	1.40, 2.34,	
				Richest	4.04	3.08, 5.30,			2.58	1.99, 3.40,	Significant
			Education								
				No Education	1				1		
				Primary	1.23	1.06, 1.43,			1	0.86, 1.17,	
				Secondary	1.68	1.49, 1.90,			1.12	0.98, 1.28,	
				Higher	1.87	1.61, 2.18,			1.01	0.86, 1.20,	NS
			In models 1–3, one SES marker (social caste, household wealth and education) was modelled at a time while adjusting for age, gender, religion and place of residence.								
			In model 5, all SES markers and BMI were included along with covariates form models 1–3.								
Sayeed et al., (1997)	High	Diabetes		Men (N=1480)	N	Diabetes (%)			Logistic regression (Poor=0)		
				Poor				Poor	ref		
				Rural	285	2.81		Rich	4.1	2.02-8.23	
				Urban	112	0.89	0.41				
				Rich							
				Rural	130	9.23					
				Urban	571	8.06	0.575				
				Middle							
				Rural	382	3.66					
				Women (N=891)							
				Poor							
				Rural	239	2.09					
				Urban	129	0					
				Rich							
				Rural	64	6.25					
				Urban	240	10.42	0.76				
				Middle							
				Rural	219	4.11					
								Final model adjusted for age, sex, BMI, waist-hip-ratio (WHR)			
Gupta et al., (2003)	High	Self-Reported Diabetes	Education					Change in diabetes between 1995 and 2002			
			(Males)		n	%			95% CI		
				Group 0 (Illiterate)	7	6.8		+6.8			
				Group I: 1-10 Years	16	8.8		+6.7	(3.1, 10.3)		
				Group Ii: 11-15 Years	16	7.9		+7.6	(4.7, 10.5)		
				Group Iii ≥ 16 Years	5	7.9		+4.8	(-0.3, 10.5)		
			Education				0.621				
			(Females)								
				Group 0 (Illiterate)	14	6.6		+6.2	(3.2, 8.5)		
				Group I: 1-10 Years	20	12.3		+5.7	(-3.4, 14.8)		
				Group Ii: 11-15 Years	12	7.3		+5.0	(-0.9, 10.9)		
				Group Iii ≥ 16 Years	3	8.3	0.995	+3.0			

Kinra et al., (2010)	High	Diabetes (Based On Blood Glucose And Self-Report)			Age standardised prevalence of diabetes	95% CI	p
			Standard of living index*:				
			Men (n=1375)				
				Low	1.8	(0.0 to 3.9)	
				Middle	3.3	(1.4 to 5.2)	
				High	8	(6.1 to 9.8)	<0.001
			Women (n=608)				
				Low	3.9	(0.8 to 6.9)	
				Middle	5.2	(2.0 to 8.5)	
				Height	5.1	(2.9 to 7.2)	0.62
					Adjusted for age		
Samuel et al (2012)	High	Diabetes (Blood Glucose Diagnosis During Treatment)	**Wealth**		Prevalence of diabetes according to quintiles of HH possessions (*Age adjusted*)	95% CI	P values for trend
			Urban (Male)	Wealth Quintile			
				1 (Lowest)	26.2	(12.3–40.0)	
				2	16.1	(8.0–23.9)	
				3	18.9	(11.8–26.2)	
				4	25.6	(17.7–33.7)	
				5 (Highest)	31.9	(25.0–38.9)	0.01
			Urban (Female)				
				1 (Lowest)	12.1	(0.0–23.9)	
				2	22.1	(12.6–31.6)	
				3	34.6	(25.3–43.9)	
				4	28.9	(19.6–38.0)	
				5 (Highest)	30.3	(22.3–37.9)	0.08
			Rural (Male)				
				1 (Lowest)	10.9	(6.3–15.7)	
				2	17.2	(11.2–23.1)	
				3	23.1	(15.6–30.8)	
				4	20.4	(12.4–28.4)	
				5 (Highest)	31.8	(19.9–43.5)	<0.001
			Rural (Female)				
				1 (Lowest)	16.1	(10.9–21.2)	
				2	15.8	(9.2–22.4)	
				3	23.3	(15.8–30.6)	
				4	27	(18.1–35.8)	
				5 (Highest)	32.1	(19.5–44.7)	0.001
			Education				
			Urban (Male)	No Education (0 Years)	15	(0.0–32.1)	
				Primary/Middle School (1–8 Years)	23.9	(17.8–30.0)	
				High/Secondary School (9–12 Years)	22.5	(17.1–27.8)	
				>12 Years Of Schooling	34.7	(24.8–44.8)	0.06
			Urban (Female)	No Education (0 Years)	31.5	(18.7–44.2)	
				Primary/Middle School (1–8 Years)	27.5	(20.9–34.0)	
				High/Secondary School (9–12 Years)	26.5	(19.6–33.5)	
				>12 Years Of Schooling	32.2	(19.9–44.4)	0.88
			Rural (Male)				
				No Education (0 Years)	25.7	(10.4–40.9)	
				Primary/Middle School (1–8 Years)	19.7	(14.3–25.1)	
				High/Secondary School (9–12 Years)	16.9	(12.6–21.1)	
				>12 Years Of Schooling	19.7	(9.0–29.9)	0.29
			Rural (Female)				
				No Education (0 Years)	19.1	(11.4–26.7)	
				Primary/Middle School (1–8 Years)	19.2	(14.6–23.7)	
				High/Secondary School (9–12 Years)	22.3	(16.3–28.4)	
				>12 Years Of Schooling	50	(20.0–79.9)	0.16

Zaman et al., (2012)	High	Diabetes (Blood Glucose Diagnosed, Self Report)	Education		Education	N with diabetes	%	p
			Men (n=1311)		Men (n=1311)		%	
				Educated	Educated	255	20.1	
				No Education	No education	106	12.4	<0.001
			Women (n=1074)		Women (n=1074)			
				Educated	Educated	138	13.1	
				No Education	No education	156	12.8	0.258
			Occupation		Occupation			
			Men (n=1311)		Men (n=1311)			
				Skilled	Skilled	102	24.1	
				Unskilled	Unskilled	186	12.9	<0.001
			Women (n=1074)		Women (n=1074)			
				Skilled	Skilled	11	12.9	
				Unskilled	Unskilled	65	7.7	0.075
			Income		Income			
			Men (n=1311)		Men (n=1311)			p trend
				High	High	186	21.2	
				Middle	Middle	84	12.2	
				Low	Low	91	16.2	<0.001
			Women (n=1074)		Women (n=1074)			
				High	High	138	15	p trend
				Middle	Middle	57	10.7	
				Low	Low	99	12.1	0.005
					NB: Age standardised			
