# Supplementary material for: A systematic review of associations between non-communicable diseases and socioeconomic status within low- and lower-middle-income countries
Source: J Glob Health. 2018 Jul 25;8(2):020409. doi: 10.7189/jogh.08.020409 (PMC6076564; doi:10.7189/jogh.08.020409)
Supplement: Online Supplementary Document [file jogh-08-020409-s001.zip › Appendix_4.4_CRD_All_Resultsv2.pdf]

**Appendix 4.4** Full results among papers reporting chronic respiratory disease

| Study | Quality | Outcome | Exposure | | Crude | % |
|---|---|---|---|---|---|---|
| Chhabra et al., (2001) | Medium | Chronic Obstructive Pulmonary Disease/ | SES (lower pollution zone) | | | |
| | | Chronic Bronchitis | | **Low SES** | | % |
| | | | | | Male | 3.1 |
| | | | | | Female | 2.1 |
| | | | | **Middle SES** | | |
| | | | | | Male | 0.8 |
| | | | | | Female | 0.7 |
| | | | | **High SES** | | |
| | | | | | Male | 1.8 |
| | | | | | Female | 0.3 |
| | | | SES (higher pollution zone) | | | |
| | | | | **Low SES** | | |
| | | | | | Male | 3.2 |
| | | | | | Female | 5.9 |
| | | | | **Middle SES** | | |
| | | | | | Male | 4.6 |
| | | | | | Female | 1.1 |
| | | | | **High SES** | | |
| | | | | | Male | 0.5 |
| | | | | | Female | 1.7 |

Note: For results by Burkart (2011) and Chandrashekar (2014), see Appendix 4.5
