# Supplementary material for: A systematic review of associations between non-communicable diseases and socioeconomic status within low- and lower-middle-income countries
Source: J Glob Health. 2018 Jul 25;8(2):020409. doi: 10.7189/jogh.08.020409 (PMC6076564; doi:10.7189/jogh.08.020409)
Supplement: Online Supplementary Document [file jogh-08-020409-s001.zip › Appendix_4.5_Multiple_All_Resultsv2.pdf]

Appendix 4.5 Full results among papers reporting multiple outcomes

Study	Quality	Outcome	Exposure	n	Crude	%	95% CI	Adjusted	95% CI	p
Binnendijk et al., (2012)	High	Self Reported NCDs								
			Education (Odisha)			%				
			Education							
			No Education			45.7				
			Class (Odisha)							
			Class 1-5			17				
			Class 6-10			29.3				
			Class 11 And Higher			6.5				
			Missing			1.5				
						mean	SD			
			Income (Odisha)							
			Income Proxy Per Person Per Month (PPP$)			30.09	0.13			
			Association between income and prevalence of illness (Odisha)*			mean				
							SE			
			Concentration Index As Described By O'Donnell (0=No Inequality)			0.157	0.019			
			Education (Bihar)			%				
			No Education			45.8				
			Class 1-5			12.2				
			Class 6-10			32.6				
			Class 11 And Higher			8.3				
			Missing			1.1				
			Income (Bihar)			mean	SD			
			Income Proxy Per Person Per Month (Papp$)			43.84	0.28			
			Association between income and prevalence of illness (Bihar)			mean				
							SE			
			Concentration Index As Described By O'Donnell (0=No Inequality)			0.115	0.016			
**A Negative index indicates a disproportionate concentration of health among the poor, zero indicates no inequality and positive indicates disproportionate concentration of health among the rich.*										
Burkart et al., (2011)	High	Percentage Of Deaths Attributable	Deaths attributable to Respiratory disease (%)							
			Low SES Region			14.10%				
			High SES Region			14.20%				0.95
			Deaths attributable to CVD							
			Low SES Region			14.70%				
			High SES Region			17.60%				<.001
			Deaths attributable to cancer							
			Low SES Region			7.10%				
			High SES Region			7.70%				0.13

Chandrashekhar et al., (2014)	Medium	Cardiovascular Disease (N) And Respiratory Disease (N)	CVD (Percentages within SES groups)	Education							
					Education Status	n (370 total)		% of people within group			<0.001
					Illiterate	285	35	12.28			
					Primary	34	11	32.35			
					Secondary	17	5	29.41			
					College	8	3	37.5			
					Graduation	22	7	31.81			
					Post-Graduate	4	4	100			
				SES	Socioeconomic Class						>0.05
					I (Upper)	5	2	40			
					Ii	22	6	27.27			
					Iii	106	21	19.81			
					Iv	133	12	11.53			
					V	104	65	17.56			
			Respiratory disease (percentages within SES groups)	Education	Education Status						
						n (370 total)					
					Illiterate	285	12	4.81			>0.05
					Primary	34	2	5.88			
					Secondary	17	0				
					College	8	0				
					Graduation	22	1	4.54			
					Post-Graduate	4	0				
				SES	Socioeconomic Class						>0.05
					I (Upper)	5	0				
					Ii	22	0				
					Iii	106	6	5.66			
					Iv	133	8	5.88			
					V	104	1	0.96			
Jerliu et al., (2013)	High	Self-Reported Chronic Diseases (Cod, Diabetes, Stomach And Liver, Lung, Neurologic Disorders, Cancer And Other Conditions)									
			Presence of chronic conditions (presence vs absence of chronic conditions)	Educational level:							
									OR	95% CI	
					≥9 Years				1	(reference)	
					1–8 Years				1.52	(0.99–2.32)	0.052
					0 Years				1.46	(0.87–2.45)	0.148
					Self-Perceived Poverty:						
					Not Poor				1	(reference)	
					Poor				1.5	(1.10–2.05)	0.01
			Multimorbidity (≥ 2 chronic conditions vs single morbidity (1 chronic condition).	Educational level:							
					≥9 Years				1	(reference)	
					1–8 Years				1.22	(0.84–1.77)	0.304
					0 Years				1.33	(0.88–2.03)	0.178
					Self-Perceived Poverty:						
					Not Poor				1	(reference)	
					Poor				1.25	(1.00–1.55)	0.049
				Adjustments:	*Sex, Age, Urban/rural, access to medical care*						

Rao et al., (2011)	Medium	Self-Reported CVD And Diabetes	CVD (including hypertension)	Education	No Schooling/Illiterate		34%				
					Primary Or Below		26%				
					Middle And Higher Schooling		31%				
					Diploma/Graduate & Above		9%	p<0.05			
			Diabetes	Education							
					No Schooling/Illiterate		27%				
					Primary Or Below		37%				
					Middle And Higher Schooling		29%				
					Diploma/Graduate & Above		7%	p<0.05			
Rossier et al., (2014)	High	Mortality From NCDs									
						n					
				**Formal neighborhoods (more affluent)**							
									Mortality rate from NCDs (per 1000 person years)		
				Male					10.4	(8.6–12.5)	
				Female					6	(4.7–7.6)	
				Education (formal neighborhoods)							
					None				11.3	(9.3–13.8)	
					Primary				5.9	(3.8–9.3)	≤.05
					Secondary Or More				4.1	(2.5–6.8)	
				Standard of living (formal neighborhoods)	Standard Of Living						
					Poor				10.6	(8.0–14.1)	
					Middle				9	(7.4–10.9)	≤.05
					Rich				4.7	(3.1–7.1)	
					All*				8.2	(7.0–9.5)	
				**Informal Neighborhoods (less affluent)**							
										95% CI	
					Male				3.6	(2.6–5.1)	
					Female				2.7	(1.7–4.5)	
				Education (informal neighborhoods)							
					None				3.8	(2.7–5.3)	
					Primary				2.7	(1.2–6.0)	
					Secondary Or More				1.7	(0.4–6.9)	NS
				Standard of living (informal neighborhoods)							
					Poor				4.1	(3.0–5.7)	
					Middle				2.1	(1.2–3.7)	
					Rich				7.1	(1.0–50.5)	≤.05
					All*				3.3	(2.5–4.3)	
Note: NCDs included CVD, Neoplasms, Other NCDs, Asthma, Diabetes, Liver Disease).					Standardised by age						

Singh et al., (2005)	High	Mortality From Cancer And Circulatory Diseases		Social class						
		Circulatory Diseases	Mortality	Males (n=1,385)		Deaths	Circulatory Diseases (%)			p
					Social Class 1 (More Affluent)	119	45			<.04
					Social Class 2	158	45.8			<.04
					Social Class 3	95	32.7			<.04
					Social Class 4	30	10.8			NS
					Social Class 5 (Less Affluent)	4	1.9			NS
					Total	406	29.3			NS
				Females (n=2,222)						
					Social Class 1 (More Affluent)	80	49			NS
					Social Class 2	108	48.8			NS
					Social Class 3	37	21.8			NS
					Social Class 4	10	6.3			NS
					Social Class 5 (Less Affluent)	5	4			NS
					Total	646	29.1			NS
		Mortality From Malignant Neoplasm	Mortality	(Males)						
						Deaths	Malignant neoplasms (%)			
					Social Class 1	16	6			NS
					Social Class 2	16	4.6			NS
					Social Class 3	19	6.5			NS
					Social Class 4	15	5.4			NS
					Social Class 5	11	5.2			
										NS
					Total	77	5.5			NS
				Females						
					Social Class 1	16	9.8			<.04
					Social Class 2	12	5.4			NS
					Social Class 3	12	7.1			NS
					Social Class 4	10	6.3			NS
					Social Class 5	4	3.2			NS
					Total	54	6.4			NS
					NB: p values obtained by comparison of SES 1-3 e.g. higher class with SES 4,5, e.g. lower class)					
Van Minh et al., (2008)	High	Self-Reported Chronic Diseases (Included Chronic Joint Problems, Heart And Circulatory Conditions, Cancer, Diabetes, Chronic Pulmonary Diseases And Psychological Illness.								
				Education (Males)				OR		NS
					Less Than Secondary School			2.4	(1.5,3.7	
					Secondary School			1	(0.8–1.5)	
					High School And Higher			1		
				SES (Males)						
					Poor			0.8	(0.6–1.6)	NS
					Middle			1.4	(1.0–1.9)	
					Rich			1		
				Education (Females)						
					Less Than Secondary School			2.7	1.7-4.3a	<.05
					Secondary School			0.6	(0.4–1.0)	
					High School And Higher			1		
				SES (Females)						
					Poor			1.4	(1.1–2.2)	<.05
					Middle			1.1	(0.8–1.6)	
					Rich			1		
					Adjusts for age, occupation, smoking and alcohol					
